# ATGC transcriptomics: a web-based application to integrate, explore and analyze de novo transcriptomic data

**DOI:** 10.1186/s12859-017-1494-2

**Published:** 2017-02-22

**Authors:** Sergio Gonzalez, Bernardo Clavijo, Máximo Rivarola, Patricio Moreno, Paula Fernandez, Joaquín Dopazo, Norma Paniego

**Affiliations:** 1 Instituto de Biotecnología, Centro Investigación en Ciencias Veterinarias y Agronómicas (CICVyA) INTA, Hurlingham, Buenos Aires Argentina; 2 Earlham Institute, Norwich Research Park, Norwich, NR4 7UG UK; 3 Consejo Nacional de Investigaciones Científicas y Técnicas (CONICET), Godoy Cruz 2290, Buenos Aires, C1425FQB Argentina; 4 Instituto de Ingeniería Biomédica, Facultad de Ingeniería, Universidad de Buenos Aires, Buenos Aires, Argentina; 5 Escuela de Ciencia y Tecnología, Universidad Nacional de San Martín, San Martín, Buenos Aires Argentina; 6 Computational Genomics Department, Centro de Investigación Príncipe Felipe, Valencia, Spain

**Keywords:** *De novo* transcriptomics, Data integration, Ontology storage, Web application

## Abstract

**Background:**

In the last years, applications based on massively parallelized RNA sequencing (RNA-seq) have become valuable approaches for studying non-model species, e.g., without a fully sequenced genome. RNA-seq is a useful tool for detecting novel transcripts and genetic variations and for evaluating differential gene expression by digital measurements. The large and complex datasets resulting from functional genomic experiments represent a challenge in data processing, management, and analysis. This problem is especially significant for small research groups working with non-model species.

**Results:**

We developed a web-based application, called ATGC transcriptomics, with a flexible and adaptable interface that allows users to work with new generation sequencing (NGS) transcriptomic analysis results using an ontology-driven database. This new application simplifies data exploration, visualization, and integration for a better comprehension of the results.

**Conclusions:**

ATGC transcriptomics provides access to non-expert computer users and small research groups to a scalable storage option and simple data integration, including database administration and management. The software is freely available under the terms of GNU public license at http://atgcinta.sourceforge.net.

**Electronic supplementary material:**

The online version of this article (doi:10.1186/s12859-017-1494-2) contains supplementary material, which is available to authorized users.

## Background

Functional genomic analysis of species lacking a reference genome involves the integration of heterogeneous data sources including transcriptome assembly and annotation, along with gene expression and genetic variants. NGS data analysis results requires the development of a user-friendly tool to manage, visualize and analyze a large amount of results in a comprehensive and integrated manner. In the last few years, many applications for conducting functional genomic studies have appeared [1, 2]. A typical *de novo* RNA-seq pipeline involves; (i) Sequence quality control (ii) Transcript assembly, (iii) Functional and structural annotation of those transcripts, (iv) Discovery of molecular markers (microsatellites (SSRs) and single nucleotide polymorphisms (SNPs), and (v) Differential expression of transcripts between the conditions assayed (See Conesa, A. et al., 2016 for a detailed review: [3]). All these steps produce large and complex structured results which need to be handled properly. Most of the applications performing these steps in a *de novo* RNA-seq pipeline greatly aided small research groups but, usually, these same groups lack data management capabilities and know how.

ATGC transcriptomics relies on the ability to store data in an orderly manner, by using an ontology-based and modular database schema such as Chado [4]. Our web application allows a broad interpretation of the produced data and the formulation of data-driven hypotheses to test biological questions. Thus, ontology-driven databases provide a flexible and adaptable schema capable of extracting complex relationships arising from the data structure. Chado is a relational database schema designed by the Generic Model Organism Database Consortium (GMOD) to handle complex representations of biological knowledge. The GMOD introduces the concept of computational ontology as applied to the management of the information structure in a way that accounts for the tacit knowledge of ontology. Computational ontologies, such as the Gene Ontology project [5] and Sequence Ontology [6], not only allow users to sort data by introducing a structure with terms and definitions, but also provide a common language to store and share information. The Chado schema, which was originally designed by FlyBase, has been widely used to develop databases of specific organisms [7, 8]. Several software packages import or export data using the Chado database schema: GBrowse [9], Maker [10], Apollo [11] and Ergatis [12].

However, the administration, management and analysis of large databases lacking a graphical interface may be complicated and non-intuitive for non-informatics users. In this context, web applications emerged as the best choice due to user’s familiarity with this kind of tools, their simple installation and shared access to databases. Recently, Tripal has appeared as a solution to develop web front-ends for Chado specifically designed to manage genomic and genetic information [13]. Tripal is a toolkit for the construction of biological, scientific research-oriented websites that is based on the Drupal content management system [14]. This toolkit provides an Application Program Interface (API) that makes the application more flexible, thus allowing complete customization of data. This option, however, requires informatics expertise, for example in a *de novo* transcriptomic assay (discussed below).

Here we present the ATGC transcriptomics, a web-based application that arose from the need for genomic data management of small research groups. In parallel, during the development process, we used this new web-based application to integrate and analyze data from different projects that involve diverse data sources [15–18].

ATGC transcriptomics is a free and open-source application available on-line designed to visualize, explore, analyze and share *de novo* transcriptomic data generated by NGS platforms using the Chado database schema to store data. The ATGC application allows users to integrate heterogeneous datasets such as structural annotation, genotype information, experimental designs, gene expression, functional annotation and other computational analyses, into a single database with a user-friendly interface. ATGC Transcriptomics has support for storing data in several modules and implements different ontologies for classification and further analysis of data through ontology-based searches, graphics, and detailed tables.

## Implementation

The ATGC application was built using the Chado database schema implemented in PostgreSQL [19], a web interface based on Web2py [20], and an in house developed Python module called pychado, which was created as an interface between the web application and the database.

The Chado schema is partitioned into modules, thus creating a scalable model with the addition of specific modules that provide support for additional data types including those from novel technologies, without modifying the actual schema. The database schema consists of five core modules, which are named Sequence, General, Publication, Audit and Controlled vocabularies (Ontologies). Moreover, ATGC transcriptomics incorporates the following modules: Organism, Companalysis, Phenotype, Strain, Genetics, Stock, and Expression, to store data from a wide variety of biological experiments and research fields, such as comparative sequence analysis, gene expression studies, genetics, taxonomy, biological collections and phenotypic diversity.

For the database interface, we used and implemented the Model – View – Controller (MVC) pattern that Web2py provides, as to access the modular schema, separating the data representation (model), data presentation (view) and application logic (controller). We used an internal Web2py database to store configuration data related to the user interface and the Chado database administration. The application appearance can be easily changed or customized since all web pages inherit styles from an application-wide cascading style sheet (CSS), and since overall page layout is controlled by a single HTML layout file to have a global control over the views. The ATGC web interface is designed to handle a variety of application tasks. Database navigation is from a set of drop-down lists including Home, Data loading, Search, Ontology annotation, Download, Software, Modify and Delete and Setup (Fig. 2a). The lists are configurable by the authorized user in a straightforward way using the Web2py administration interface.

The pychado module makes the connection of the application with the Chado database using the psycopg python package [21]. Pychado contains a structure of data classes and methods to insert, modify or delete data entries and get information adapted to the Chado modules and tables. Furthermore, taking into account the ontology-driven storage, we worked in modeling ontology terms and structures to go through the ontology graph to save data and enable searches and queries.

ATGC transcriptomics permits the storage of sequence-related data, including structural annotation (GFF format), sequence alignments obtained by BLAST [22], functional annotation results using Blast2GO and InterProScan and classification of other database features using any other ontology that can be loaded (tabular file format). This application allows users to load data from genotypes or strains, genetic variants, molecular markers, sequence relationships (e. g., assembly of gene isoforms). Moreover, it is also possible to load and analyze diverse experimental approaches. For example, users can load differential gene expression studies, including the assay structure with replicates and the experiment characteristics specified using ontological classification [23].

For small scale projects, a typical desktop computer can be used to install and run ATGC transcriptomics. The application was tested and works correctly in a virtual machine with 1 CPU and 1Gb of RAM and requires an average of 500 Mb of RAM together with the Linux operating system [24]. The complete installation from source code requires a Unix operating system (e. g., Linux) with a minimum set of software packages and a single administration user with basic UNIX skills (see manual). Additionally, a pre-installed self-contained virtual machine is available. Users using any operating system (e. g., Windows) may download the complete virtual machine to bypass the installation steps. Detailed instructions for different types of installation are provided in Additional files 1 and 2.

**Fig. 1:**
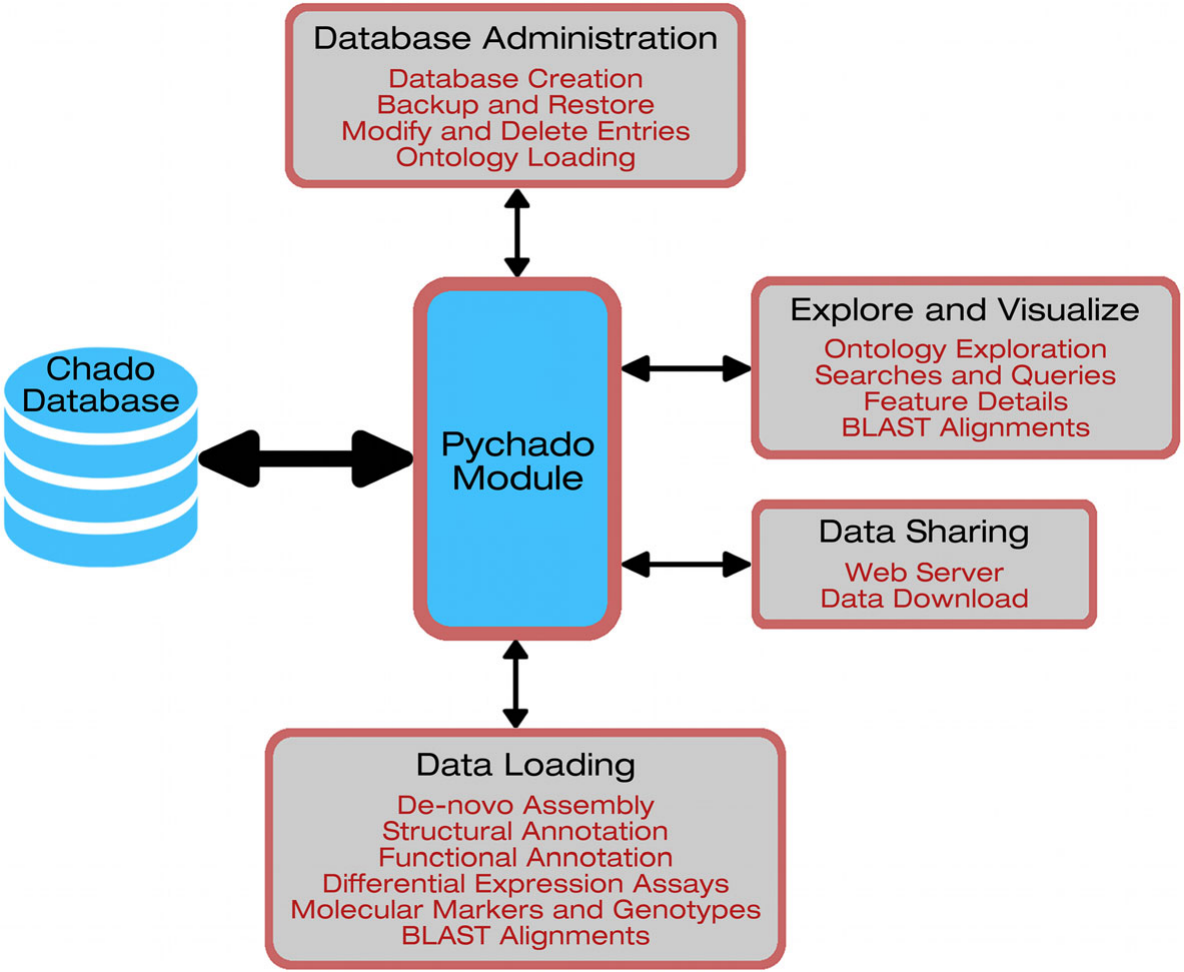
Application schema. Schema illustrating how the four main components interact with the Chado database through the pychado module. Each component contains a set of functions (controllers) and views which enable users to access to the database content

ATGC transcriptomics is structured in four main components that are accessible through the web browser: The components are (1) database administration and management, (2) data loading and results of software analysis, (3) searches and queries, and (4) data sharing (Fig. 1).

### Database administration and management

We developed an interface for database creation, administration, update, backup, restore and ontologies loading. We generated a template for database schema in SQL format, with all the modules to be used including small modifications to load RNA-Seq data (not fully supported in Chado). The interface allows users to load ontologies using files in OBO and XML format and also load data with relationships between ontologies and several classification systems, in a section called dbxref2ontology (i. e. Interpro2go). ATGC transcriptomics allows user to have several databases on the same application instance, with the option to switch between them and maintain the original environment settings automatically. Moreover, it is possible to keep the information updated and curated in the database by using the modify and delete section. These processes take into account data types and the database schema. Furthermore, the backup and restore components allow users to create a complete database backup in SQL format. For this reason, the user can restart the database to a previous state when required.

### Load data and results of software analysis

The application is capable of loading data to the Chado database using standard file formats and of adding information one at a time or with bulk loader. ATGC implements loading methods for different types of sources and software results, such as FASTA format for sequences, GFF for structural annotations, XML for BLAST results, ANNOT or RAW for Blast2GO or InterProScan functional annotations and VCF for molecular markers and genetic variants. Moreover, several outputs from specific software (i. e. misaSSR [25] or ssahaSNP [26]) and simple file formats (generally CSV or TAB) are implemented to load additional information (i.e., relationships between features). The CSV or TAB files can be created from flat text files using a web interface, such as Galaxy [27], or exporting directly spreadsheets. From the Chado stock and genotype modules, we included the concept of genotype (line) with its characteristics that are associated and defined by ontology terms. As a consequence, we give the possibility to load the alleles of different types of molecular markers to each genotype. Finally, using the Chado modules: expression, stock, and library, we created an accessible way to load the complete structure of RNA-Seq experiments. The load includes the characteristics of the conditions defined by the ontology terms and the experimental design with replicates. The user can load different measured values to a sequence in a replicate-specific manner from a particular experimental condition, including genotype identity, transcript expression values measured as reads per kilobase of a million of mapped reads (RPKM), among others. Detailed information on all steps in load data and database administration are in the Additional file 2.

### Searches and query

**Fig. 2:**
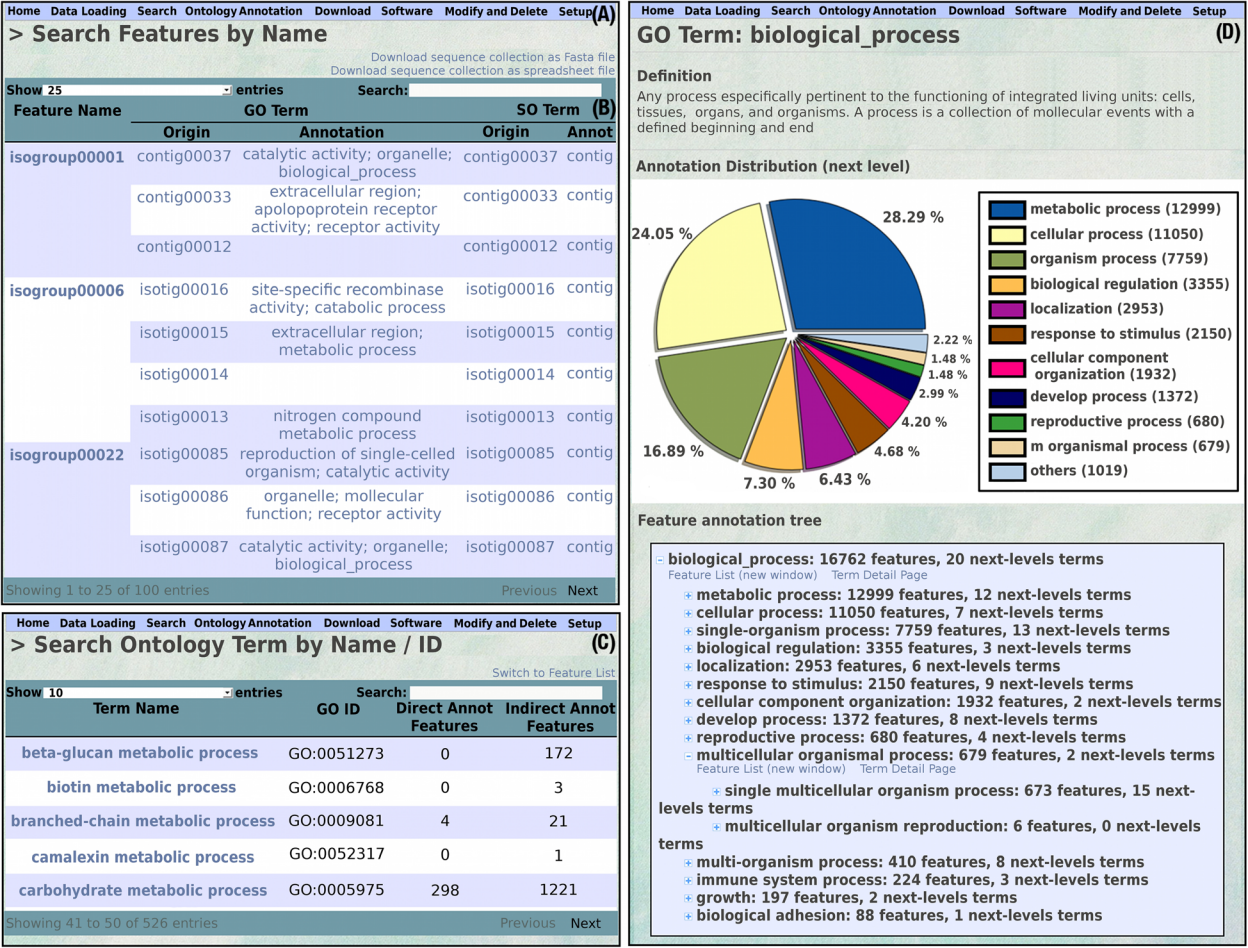
Screenshot of ATGC views. **a** Drop down menu displaying all available options. **b** Search output by gene name. The Table shows the transcript inferred functional annotation. Tools to define Table setting and internal searches are provided at the Table header. **c** Search output by ontology terms; the Table displays direct and indirect annotated features and contains a link to deploy the members associated to the feature names. **d** Pie charts and structured graph to explore annotation from any loaded ontology. The pie chart shows the distribution of annotation in direct children terms. The sequence counts associated to each classification take into account the possible multiple annotations; users have links to obtain the lists of sequences annotated under each term

The application supports different options of data searching and querying. On the one hand, the user can browse data with a text search (optionally using regular expressions, similar to SQL programing language). ATGC queries include unique feature names, lists of feature names, ontology terms (Fig. 2c) or descriptions retrieved from BLAST hits. Figure 2b shows a search output obtained using a gene name. The result is summarized in a user friendly table containing the gene characteristics and the functional annotation from all isoforms transcribed from the gene. On the other hand, the user can explore the data from the ontologies using pie charts and a graph-structured view (Fig. 2d). It is possible to move through the ontology for exploring the features annotated in each term, taking into account the ontology structure and the relationships between terms. We implemented an algorithm for navigating through ontology graphs, thus reducing the amount of RAM memory used. We merged the two ways of data exploration, to allow users to search and query the result table. They also have the option to explore the ontology pie chart and the graph structured view solely for the features present in the result table. Moreover, BLAST searches can be performed using external sequences as query and database features as subjects.

**Fig. 3:**
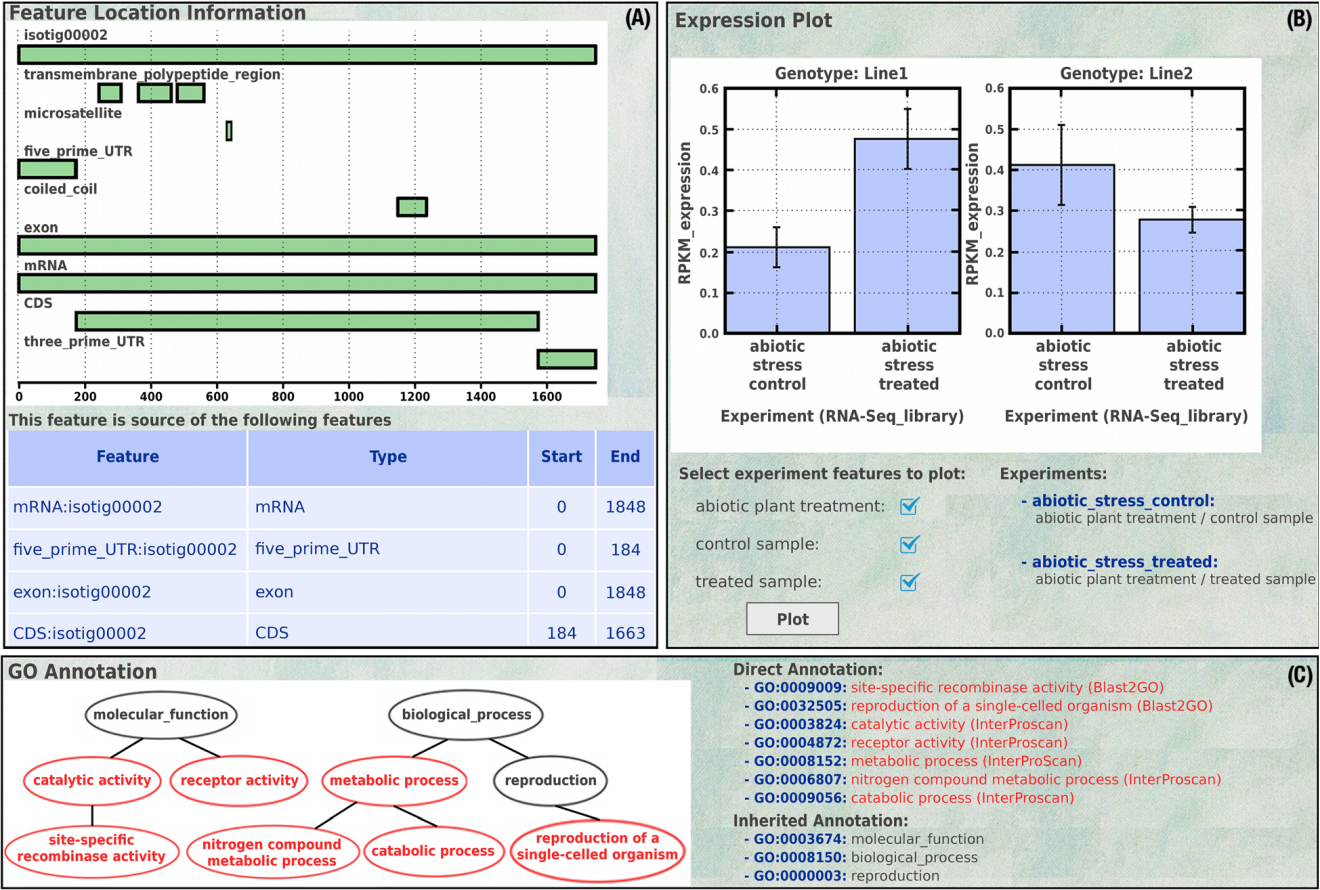
Feature details. **a** Graphic representation of a transcript with a detailed description of exons, CDS, mRNA, UTRs, molecular markers and protein domains. The table below the graphic shows the features relationships with reference positions. **b** Dynamic expression graphics, taking into account the experiment structure; users can select the conditions to plot and generate graphics divided by statistical value and genotype. The charts display the mean and standard deviation values of replicates in each condition. **c** GO acyclic graph shows functional annotation terms related to the feature annotations. The lists contain Direct and Inherited associated ontology terms with links to detailed information about each term

Regarding the result table view, the user has several options to display the feature details depending on the feature type. We worked on four display formats including sequence, marker, experiment, and genotype. The sequence report has a general and a detail view. The general report only has a relationship section ready for features without nucleotide sequence. The detail sequence report comprises six sections containing the nucleotide sequence, non-positional relationships, structural annotation, functional annotation, Blast results, and gene expression results. The nucleotide sequence has a download option in FASTA format. The relationships section contains a table that describes connections with other features (e. g., gene from which a sequence is transcribed). The structural annotation is displayed in a graphical browser and uses a detailed table (Fig. 3a). The functional annotation is represented by the ontology terms on a reduced ontology graph showing only terms and connections involved in the annotation of the specific sequence (Fig. 3c). The BLAST results are shown in a detailed table including cross references to NCBI databases. The expression profiles are represented using dynamic figures obtained from the experiment structure and separated by genotypes and statistic values (Fig. 3b). The detail marker report contains the location of the marker and the alleles of the marker on different genotypes. Finally, the experiment report includes the experiment design with libraries and experimental conditions and the genotype report has the genotype characteristics and the alleles of all the reported markers.

### Data sharing

Regarding data sharing, the users can share information with colleagues (external system users) with a simple machine or server, by using the application through the network connection with the Apache HTTP server [28], or directly with the web server included in Web2Py framework. The users can adapt the drop-down menu to show the chosen options and hide the parts of the menu where they can modify the database. Optionally, with small modifications the users have the choice to use the Web2py access control mechanism to manage the permissions for the external users to perform restricted operations (i.e., restricting access to database modifications keeping the options in the drop-down menu). Furthermore, we created several points for data access, inside the download section (general files, such as complete functional annotation in ANNOT format) or particular downloads in other places (e. g., Download FASTA sequence in the sequence report page or spreadsheets for search result tables).

## Results and discussion

In the last years, the use of NGS in biological projects has enhanced the needs for data management and storage. Several researchers have adopted Chado database to integrate sequence data, annotations, publication, strains, genotypes and several other data types and they have also created web-accessible databases to manage and analyze their data [29–31]. Simultaneously, different tools to manage genomic information providing an interface to Chado databases have become available. One example is the GMODWeb [32]. This tool is based on a MVC web framework based on Perl for the construction of online websites called TurnKey [33]; however, it is no longer supported by the GMOD project. ChadoOnRails is another framework [34] that provides a Ruby on Rails interface to help developers create the web (or other) interfaces into Chado, but does not provide data loaders and view pages. Badger [35] is a genome exploration environment implemented using the Grails web application framework [36] mainly oriented to explore genomic data. GMODWeb and ChadoOnRails require informatics expertise and need to develop some views and interphases. Today, the most popular tool for this purpose is Tripal, which provides an interface that extends the content management features of Drupal to the data housed in Chado. This application has a web-based database installer, data loaders, and extensions that support to basic data visualization. Tripal is specifically designed to manage genomic and genetic information but not all the Chado modules are integrated. For instance, Tripal does not allow working with expression related data, because the Chado expression module is not incorporated into the default templates provided [37]. Moreover, Tripal lacks an “out of the box tool” with easy installation and pre-configured framework for *de novo* RNA-seq projects (Table 1). In this sense, ATGC constitutes the single Chado framework to integrate transcriptomic data with expression studies taking into account the genomic and genetic context.

**Table 1:** Tools capability comparison

		ATGC	Tripal
Complete software installation		✓✓✓	✓
Access to personalize whole application		✓	✓✓
Access control for external (non-administrative) users		✓✓	✓✓✓
Out of the box ready querying of ontology terms (GO, SO) in database		✓✓✓	✓
Expression data integrated in schema (ready-to-use)		✓✓✓	Ni
Functional Annotation Loading	Blast2Go (annot files)	✓✓✓	✓
	InterProScan (raw or xml files)	✓✓✓	✓✓✓
	Kegg Pathways (KAAS output files)	Ni	✓✓✓

GMODWeb, ChadoOnRails, and Badger cannot be included in the comparison because could not be installed and tested for lack of documentation or support. ✓ - computer expertise required; ✓✓ - moderately easy to complete; ✓✓✓ - straightforward; *Ni* Not implemented

### De novo RNA-seq case study

We will test the ATGC transcriptomics application and will show different ways to respond to several biological questions arising from RNA-Seq data. For this purpose, we will use an training dataset from the transcriptome sequencing and differential expression assay of Diachasmimorpha Longicaudata [18]. Three conditions are analyzed (male-larvae, adult females and adult males). The main objective of this project was to identify new transcripts involved in the sex determination mechanism and develop new molecular markers for those transcripts to study them in natural populations. The data consist of reads obtained from 454 sequencing, assembled into “isotigs” (transcript contigs) with the Newbler assembler [38]. Moreover, specifically in this project, we ran a battery of applications to our original dataset to obtain the following: (1) Structural Annotation of all transcripts: using Transdecoder [39]; (2) Functional Annotation: the Blast2Go suite and the InterProScan software; (3) SSR discovery: the misaSSR software; (4) SNP discovery: ssahaSNP tool; and (5) Transcript Expression: relative expression level for a given transcript was calculated normalizing the read counts by the length of that transcript and divided by the number of sequence reads in the library.

Firstly, we create a new database and load the ontologies the user specifies (GO and SO are mandatory). In this case, they will be the GO, SO, and the hymenoptera anatomy ontology (HAO) [40]. We load the data using the web interface as explained in the tutorial. We create an organism and then we load the contigs (“*isotigs*” in *Newbler* transcriptomic nomenclature) using a FASTA file and the genes (“*isogroups*” by Newbler) using the section “Load Features from list file”. We proceed in this way, since we do not have sequences for these “*isogroups*” (a type of gene locus: the assembler provides an inferred relationship between “*isotigs*”). Next, we load the structural annotation using “Load features and relationships from gff3 file”, the functional annotation using “Feature -> CV associations”, and the Blast results used for the annotation with the section “Blast Run Results (XML Files)”. Molecular markers in VCF format (SNPs and SSRs) are loaded using “Load markers”. To load the differential expression data, first, we create the structure of experiments and libraries. We load a set of three experiments using the section “Create Experiment” called: “male_larvae”, “male_adult”, and “female_adult” with one RNA-Seq library for each. To assign the features to describe the experiments (for example phenological state and sex), we use the hymenoptera anatomy ontology. Finally, we load the expression data using a read pseudocount values for each contig using the section “Feature -> Library associations”.

After all information has been saved properly, we can begin to query the database. The first question that was raised in this project was which contigs have functional annotation related with “GO:0003006: developmental process involved in reproduction” as well as microsatellites markers associated, and which was the expression of these transcripts among the three experimental conditions. To achieve this, the user goes to the section “Search -> Features by name” and obtain a result table with all microsatellites stored in the database, using only ‘%’ as search expression in the microsatellite pull down menu (Additional file 3). Afterwards, the simplest way for the analysis is to navigate using the GO exploration at the bottom of the page where you will be able to navigate all GO terms associated with the list created above. Hence, you can explore your way to any GO term using the full capacity of the controlled vocabulary. When the user reaches the GO Term: “developmental process involved in reproduction”, he can click in “Feature List (new window)” and explore those transcripts with that GO term and at least have one SSR associated (Additional file 4). From a total of 982 transcripts with at least one SSR, we find that 8 transcripts have the GO:0003006 associated. In each detailed view of each transcript the user can examine the expression patterns of that transcript in all conditions. Exploring the “Feature report” of the transcripts, you found one “*isogroup*” with three “*isotigs*” which are highly expressed in the larvae stage and not in adult (male or female). This putative “*isogroup*” (gene locus) is probably one of the target to further investigate.

## Conclusions

In brief, this application provides non-expert computer users with accessible biological data management and simple data integration, as it can be used without any prior knowledge of programming, database administration, and/or management. ATGC transcriptomics presents a more comprehensive interface for ontological storage and browsing as a method to explore information and relationships between transcripts and other features of interest, such as the concept of alternative splicing, and relationships between transcripts and genes (or genomic loci) with functional annotation. ATGC offers the opportunity to expand the database schema by adding other modules that store information from different data sources (i.e., microarrays, phylogeny, and genetic maps). Moreover, the web-interface can easily be improved via Python modules that can be automatically copied and distributed with the application. Overall, ATGC is a user-friendly application which allows small research groups to handle their *de novo* transcriptome data as a whole.

## Availability and requirements

**Project name**: ATGC transcriptomics.

**Project home page**: http://atgcinta.sourceforge.net.

**Project demo site**: http://atgc-sur.inta.gob.ar.

**Operating system(s)**: Platform independent, source code installation or using complete virtual machine.

**Programming language(s)**: Python.

**Software requirements**: Postgresql, Ncbi-blast+, Emboss, Python, Python packages: Matplotlib, Pygraphviz, Biopython, Psycopg2, Perl, Perl packages: Bioperl, Libgo, Libpg, Libdata-stag, Libdbix-dbstag, Libsql-translator and Apache2 (Optional).

**License**: ATGC transcriptomics is freely available under the terms of the GNU public license.

## Additional files

Additional file 1: An installation guide for ATGC. (TXT 4 kb)
Additional file 2: User’s guide for ATGC (PDF 16572 kb)
Additional file 3: Search strategy to obtain the complete list of microsatellites and results table containing isotigs associated to each molecular marker and your functional annotation. Below the table there are a three links to explore gene ontology information of the isotigs in the table. (JPG 9360 kb)
Additional file 4: Pie chart of “biological_process” GO term from links in Additional file 3. In the feature annotation tree, we explore until arrive to “developmental process involved in reproduction” term and go to obtain the list of features annotated with this term. (JPG 8198 kb)

## Abbreviations

API: Application program interface
BLAST: Basic local alignment search tool
CSS: Cascading style sheet
CSV: Comma-separated values
GFF: General feature format
GMOD: Generic model organism database consortium
HTML: HyperText markup language
HTTP: Hypertext transfer protocol
MVC: Model-view-controller
NCBI: National Center for Biotechnology Information
NGS: Next generation sequencing
OBO: Open biomedical ontologies
RAM: Random access memory
RPKM: Reads per kilobase of a million of mapped reads
SQL: Structured query language
VCF: Variant call format
XML: Extensible markup language

## References

[1] Conesa A, Götz S, García-Gómez JM, Terol J, Talón M, Robles M (2005). Blast2GO: a universal tool for annotation, visualization and analysis in functional genomics research. Bioinformatics.

[2] Zdobnov EM, Apweiler R (2001). InterProScan - an integration platform for the signature-recognition methods in InterPro. Bioinformatics.

[3] Conesa A, Madrigal P, Tarazona S, Gomez-Cabrero D, Cervera A, McPherson A, Szcześniak MW, Gaffney DJ, Elo LL, Zhang X, Mortazavi A (2016). A survey of best practices for RNA-seq data analysis. Genome Biol.

[4] Mungall CJ, Emmert DB (2007). A Chado case study: an ontology-based modular schema for representing genome-associated biological information. Bioinformatics.

[5] Ashburner M, Ball CA, Blake JA, Botstein D, Butler H, Cherry JM, Davis AP, Dolinski K, Dwight SS, Eppig JT, Harris MA, Hill DP, Issel-Tarver L, Kasarskis A, Lewis S, Matese JC, Richardson JE, Ringwald M, Rubin GM, Sherlock G (2000). Gene ontology: tool for the unification of biology. The Gene Ontology Consortium. Nat Genet.

[6] Eilbeck K, Lewis SE, Mungall CJ, Yandell M, Stein L, Durbin R, Ashburner M (2005). The Sequence Ontology: a tool for the unification of genome annotations. Genome Biol.

[7] Tweedie S, Ashburner M, Falls K, Leyland P, McQuilton P, Marygold S, Millburn G, Osumi-Sutherland D, Schroeder A, Seal R, Zhang H (2009). FlyBase: enhancing Drosophila Gene Ontology annotations. Nucleic Acids Res.

[8] Jung S, Lee T, Ficklin S, Yu J, Cheng C, Main D. Chado use case: storing genomic, genetic and breeding data of Rosaceae and Gossypium crops in Chado. Database. 2016;2016:baw010. 10.1093/database/baw010 PMC4795932 26989146

[9] Donlin MJ (2009). Using the Generic Genome Browser (GBrowse). Current Protocols in Bioinformatics.

[10] Holt C, Yandell M (2011). MAKER2: an annotation pipeline and genome-database management tool for second-generation genome projects. BMC Bioinformatics.

[11] Lewis SE, Searle SMJ, Harris N, Gibson M, Lyer V, Richter J, Wiel C, Bayraktaroglu L, Birney E, Crosby MA, Kaminker JS, Matthews BB, Prochnik SE, Smithy CD, Tupy JL, Rubin GM, Misra S, Mungall CJ, Clamp ME. Apollo: a sequence annotation editor. Genome Biol. 2002;3:RESEARCH0082. 10.1186/gb-2002-3-12-research0082 PMC151184 12537571

[12] Orvis J, Crabtree J, Galens K, Gussman A, Inman JM, Lee E, Nampally S, Riley D, Sundaram JP, Felix V, Whitty B, Mahurkar A, Wortman J, White O, Angiuoli SV (2010). Ergatis: a web interface and scalable software system for bioinformatics workflows. Bioinformatics.

[13] Ficklin SP, Sanderson L-A, Cheng C-H, Staton ME, Lee T, Cho I-H, Jung S, Bett KE, Main D. Tripal: a construction toolkit for online genome databases. Database. 2011;2011:bar044. 10.1093/database/bar044 PMC3263599 21959868

[14] Drupal content management system. http://www.drupal.org

[15] Helguera M, Rivarola M, Clavijo B, Martis MM, Vanzetti LS, González S, Garbus I, Leroy P, Šimková H, Valárik M, Caccamo M, Doležel J, Mayer KFX, Feuillet C, Tranquilli G, Paniego N, Echenique V (2015). New insights into the wheat chromosome 4D structure and virtual gene order, revealed by survey pyrosequencing. Plant Sci.

[16] Torales SL, Rivarola M, Pomponio MF, Gonzalez S, Acuña CV, Fernández P, Lauenstein DL, Verga AR, Hopp HE, Paniego NB, Poltri SNM (2013). De novo assembly and characterization of leaf transcriptome for the development of functional molecular markers of the extremophile multipurpose tree species Prosopis alba. BMC Genomics.

[17] Fernandez P, Soria M, Blesa D, DiRienzo J, Moschen S, Rivarola M, Clavijo BJ, Gonzalez S, Peluffo L, Príncipi D, Dosio G, Aguirrezabal L, García-García F, Conesa A, Hopp E, Dopazo J, Heinz RA, Paniego N. Development, characterization and experimental validation of a cultivated sunflower (Helianthus annuus L.) gene expression oligonucleotide microarray. PLoS One. 2012;**7**:e45899. 10.1371/journal.pone.0045899 PMC3482228 23110046

[18] Mannino MC, Rivarola M, Scannapieco AC, González S, Farber M, Cladera JL, Lanzavecchia SB (2016). Transcriptome profiling of Diachasmimorpha longicaudata towards useful molecular tools for population management. BMC Genomics.

[19] PostgreSQL. http://www.postgresql.org

[20] Web2py. http://www.web2py.com

[21] Psycopg python package. http://initd.org/psycopg/

[22] Altschul SF, Gish W, Miller W, Myers EW, Lipman DJ (1990). Basic local alignment search tool. J Mol Biol.

[23] Mungall CJ, Batchelor C, Eilbeck K (2011). Evolution of the Sequence Ontology terms and relationships. J Biomed Inform.

[24] LUbuntu. http://lubuntu.net/

[25] Thiel T, Michalek W, Varshney RK, Graner A (2003). Exploiting EST databases for the development and characterization of gene-derived SSR-markers in barley (Hordeum vulgare L.). Theor Appl Genet.

[26] Zemin N, Caccamo M, Mullikin JC (2005). ssahaSNP a polymorphism detection tool on a whole genome scale. 2005 IEEE Computational Systems Bioinformatics Conference - Workshops (CSBW’05). IEEE.

[27] Goecks J, Nekrutenko A, Taylor J (2010). Galaxy: a comprehensive approach for supporting accessible, reproducible, and transparent computational research in the life sciences. Genome Biol.

[28] Apache HTTP server. http://apache.org/

[29] Droc G, Lariviere D, Guignon V, Yahiaoui N, This D, Garsmeur O, Dereeper A, Hamelin C, Argout X, Dufayard J-F, Lengelle J, Baurens F-C, Cenci A, Pitollat B, D’Hont A, Ruiz M, Rouard M, Bocs S. The Banana Genome Hub. Database. 2013;2013:bat035. 10.1093/database/bat035 PMC3662865 23707967

[30] Yu J, Jung S, Cheng C-H, Ficklin SP, Lee T, Zheng P, Jones D, Percy RG, Main D (2014). CottonGen: a genomics, genetics and breeding database for cotton research. Nucleic Acids Res.

[31] Ficklin SP, Feltus FA (2013). A systems-genetics approach and data mining tool to assist in the discovery of genes underlying complex traits in Oryza sativa. PLoS One.

[32] O’Connor BD, Day A, Cain S, Arnaiz O, Sperling L, Stein LD (2008). GMODWeb: a web framework for the Generic Model Organism Database. Genome Biol.

[33] Turnkey. http://genome.ucla.edu/turnkey/

[34] Chado on rails framework. http://gmod.org/wiki/Chado_on_Rails

[35] Elsworth B, Jones M, Blaxter M (2013). Badger - An accessible genome exploration environment. Bioinformatics.

[36] Grails. http://grails.org

[37] Sanderson L, Ficklin SP, Cheng C, Jung S, Feltus FA, Bett KE, Main D. Tripal v1.1: a standards-based toolkit for construction of online genetic and genomic databases. Database. 2013;2013:bat075. 10.1093/database/bat075 PMC3808541 24163125

[38] Newbler. http://www.454.com/products/analysis-software/

[39] Haas BJ, Papanicolaou A, Yassour M, Grabherr M, Blood PD, Bowden J, Couger MB, Eccles D, Li B, Lieber M, Macmanes MD, Ott M, Orvis J, Pochet N, Strozzi F, Weeks N, Westerman R, William T, Dewey CN, Henschel R, Leduc RD, Friedman N, Regev A (2013). De novo transcript sequence reconstruction from RNA-seq using the Trinity platform for reference generation and analysis. Nat Protoc.

[40] Yoder MJ, Mikó I, Seltmann KC, Bertone MA, Deans AR (2010). A gross anatomy ontology for hymenoptera. PLoS One.

