# Supplementary material for: ATGC transcriptomics: a web-based application to integrate, explore and analyze de novo transcriptomic data
Source: BMC Bioinformatics. 2017 Feb 22;18:121. doi: 10.1186/s12859-017-1494-2 (PMC5320735; doi:10.1186/s12859-017-1494-2)
Supplement: Additional file 2: — User’s guide for ATGC (PDF 16572 kb) [file 12859_2017_1494_MOESM2_ESM.pdf]

ATGC Application User Manual

Table of Contents

1 Description....4
2 Install application....4
  2.1 System Requirements....4
  2.2 Complete instalation (without vitual machine)....4
    2.2.1 Software requirements....4
    2.2.2 PostgeSQL configuration....6
  2.3 Uncompress application file....6
  2.4 Instalation using Virtual machine....6
    2.4.1 VirtualBox install....6
    2.4.2 Import VM Image....7
3 Runing application (Using Web2Py executable)....7
  3.1 Using screen command: 7
  3.2 Using background (choose a password):....7
  3.3 Using the graphical user interface (GUI): 7
  3.4 Using web2py http server....7
  3.5 Using Apache web server 7
4 Application access using the web browser....8
5 Application Usage....9
  5.1 Database creation and load ontologies (Setup menu)....9
    5.1.1 Database creation....9
    5.1.2 Database selection....10
    5.1.3 Load Ontologies....10
    5.1.4 Dump and Restore....10
  5.2 Data loading 11
    5.2.1 Create Organism....11
    5.2.2 Load features from fasta....11
    5.2.3 Features → CV associations (load cvterms for features) 12
    5.2.4 Blast run results (XML Files)....12
    5.2.5 Genotypes (lines), markers and alleles....13
    5.2.6 Load expression information....15
    5.2.7 Feature relationships....17
  5.3 Sequence search....18
    5.3.1 Search features by name....18
    5.3.2 Search features by list of names....19
    5.3.3 Search features by ontology term name or accession....20
    5.3.4 Search by Blast matches....21
  5.4 Ontology exploration....23
  5.5 Download....24
  5.6 Software....25
    5.6.1 Blast....25
  5.7 Modify and delete....27
6 Share information....29
7 Testing the application....33

Table of Figures

Figure 1: First step in application......9
Figure 2: Database creation......9
Figure 3: Database selection......10
Figure 4: Load ontologies......10
Figure 5: Data loading after create an organism......11
Figure 6: Load features from fasta file......11
Figure 7: Feature → CV associations......12
Figure 8: Load Blast results......12
Figure 9: Create new line......13
Figure 10: Lines → CV associations......13
Figure 11: Lines → CV associations......13
Figure 12: Load markers......14
Figure 13: Markers → Line associations......14
Figure 14: Create experiment......15
Figure 15: Create library associatted to a line and experiment......15
Figure 16: Experiments → CV associations......15
Figure 17: Experiments → CV associations (second part)......16
Figure 18: Feature → Library associations......16
Figure 19: Load relationships between features......17
Figure 20: Load features relationships from gff3 file......17
Figure 21: Search by name......18
Figure 22: Search by name result......18
Figure 23: Search list of features by name......19
Figure 24: Search features by ontology annotation......20
Figure 25: Search by ontology, summary result......20
Figure 26: Search by ontology, feature list result......21
Figure 27: Search features by Blast matches......21
Figure 28: Search by Blast matches results......22
Figure 29: Pie chart: Biological process......23
Figure 30: Feature annotation tree (Dropdown menu)......24
Figure 31: Download feature file......24
Figure 32: Download annotations......24
Figure 33: Create Blast database......25
Figure 34: Run Blast alignments......25
Figure 35: Example of Blast alignment......26
Figure 36: Example of Blast result......26
Figure 37: Search object to modify......27
Figure 38: Table to select object to modify......27
Figure 39: Update form to modify values......28
Figure 40: Web2py administration interface: write a password......29
Figure 41: Choosing "Edit" option......30
Figure 42: Click on "database administration" button......31
Figure 43: Click on db.navbar option......31
Figure 44: Hide the "Setup" part of the menu......32

Index of Tables

Table 1: Id values for main menu bar options in db.navbar table..32

1 Description

ATGC is a web application that allows users to work with NGS transcriptomic data without a reference genome, using an ontology driven database schema to data store and management and provide interfaces to create schema structure and load ontologies and data, visualization, searching possibilities and data integration. Using this application is possible visualize, explore, analyze and share de novo transcriptomic data generated by NGS platforms using a Chado database to store the data. It is an open source and free available application, with support to store information in several Chado modules and uses different ontologies to classify data and then explore this data using a ontology structure with graphics, searches and description tables. ATGC is open source, so all we ask is that you cite our paper in any publications that use this application:

Cita

ATGC is a open source application, for more information, new releases, download source code and complete virtual machines (see below) or download the manual please visit the homepage: **http://atgcinta.sourceforge.net**

ATGC is available for installing in two ways, the first is a complete instalation mode (only tested for Linux machines) or the second is the use of virtual machines, downloading a VM image with the complete application (all requirements installed there). For detailed installation instructions please view the next points of this manual.

2 Install application

The application is dowloadble from: **http://atgcinta.sourceforge.net**, from this URL is possible to download source code and virtual machine (VM) images.

2.1 System Requirements

The software have been tested in the following Operative Systems:

- Distributions of Linux derived from Debian (Extensively tested in Debian7.0/5.0, Ubuntu12.04/14.04/15.04/16.04)
- Distributions of Linux derived from Red-Hat (Tested in Fedora20)
- For Windows or Mac (if you want also in any Linux distribution) please view the details below.

The application was tested (and run correctly) in a machine with:

- 1 CPU
- 1 Gb RAM (Using 500 Mb in average)

2.2 Complete instalation (without vitual machine)

2.2.1 Software requirements

The ATGC application require the following to run succesfully. In the absence of one or more of these packages, some ATGC parts may fail to run correctly. Listed in parenthesis are the versions used to test the application. These versions, or subsequent versions should assure the proper execution. These utilities must be accessible via the system path:

To install in Linux Debian (or related distributions), packages required:

- postgresql (9.3.5-0)
- ncbi-blast+ (2.2.28-2)
- emboss (6.6.0-1)
- python (2.7)
- python packages:
    - python-matplotlib (1.3.1-1)
    - python-pygraphviz (1.2-1)
    - python-biopython (1.63-1)
    - python-psycopg2 (2.4.5-1)
- perl (5.18.2)
- perl packages:
- perl-doc (3.19)
    - bioperl (1.6.923-1)
    - libgo-perl (0.15-1) (After install this package please change in the script: /usr/bin/go2chadoxml: go2fmt.pl to go2fmt)
    - libpg-perl (1:2.1.1-4)
    - libdata-stag-perl (0.13-1)
    - libdbix-dbstag-perl (0.12-1)
    - libsql-translator-perl (0.11018-1)

To install in Linux Red-Hat (or related distributions), packages required:

- postgresql-server (9.3.5-1)
- postgresql-contrib (9.3.5-1)
- postgresql-devel (9.3.5-1)
- ncbi-blast+ (2.2.29-2)
- EMBOSS (6.4.0-11)
- python27 (2.7)
- python packages:
    - python-matplotlib (1.3.1-3)
    - graphviz-python (2.34.0-8)
    - python-biopython (1.64-1)
    - python-psycopg2 (2.5.1-2)
- perl (5.18.2)
- perl packages:
    - perl-doc (3.20)
    - bioperl (1.6.924)
    - go-perl (0.15)
    - PgSQL (0.51)
    - Data-Stag (0.14)
    - DBIx-DBStag (0.12)
    - SQL-Translator (0.11020)

2.2.2 PostgeSQL configuration

The application need to create and enable the user with which are running in the postgresql package. For this, execute the following commands replacing <username> by the correct name.

$ su -

$ su - postgres

$ createuser <username>

Enter to postgresql to enable the user to database creation:

$ psql

$ ALTER ROLE <username> CREATEDB;

$ exit

2.3 Uncompress application file

To uncompress the application run the following command:

tar xzvf ATGC-1.0_source.tar.gz

This file contains a directoy called web2py and include all files to run the application.

For the specific files for ATGC application, you must go to **<path to>/web2py/applications/ATGC**

2.4 Instalation using Virtual machine

For the instalation using the virtual machine (VM) image and VirtualBox software you can download the VM image from **http://www.mediafire.com**

This VM has all packages installed and is avaliable in 32 and 64 bits.

The VM has the following features:

- LUbuntu16.04
- 1 CPU
- 1 Gb RAM
- 100 Gb Hard Drive
- username: atgc
- password: atgc
- root password: atgc

2.4.1 VirtualBox install

VirtualBox software can be downloaded and install from: **http://www.virtualbox.org/wiki/Downloads** Choose the correct package according to your operating system.

2.4.2 Import VM Image

To import VM image download and decompress **ATGC_XXb.ova (XX = 32 or 64)** file from:

http://www.mediafire.com/file/0bbocuj5uvcjc6o/ATGC_32b.ova

or

http://www.mediafire.com/file/dsx3evld8w5y1a6/ATGC_64b.ova

Open VirtualBox :

- **File -> Import Appliance (Use the .ova file to import)**

3 Runing application (Using Web2Py executable)

The password is needed when the user want to access to the administrative interface.

For the virtual machine mode installation replace <path to> by /home/atgc, for complete instalations replace by the correct path.

3.1 Using screen command:

$ screen -S web2py

$ <path to>/web2py.py --nogui

3.2 Using background (choose a password):

$ <path to>/web2py.py --nogui --password=password &

3.3 Using the graphical user interface (GUI):

$ <path to>/web2py.py

3.4 Using web2py http server

$ screen -S web2py

$ <path to>/web2py.py --nogui --interface='localhost:8000;<serverIP>:<port>'

In this way, other machines in the network can access to the application, writing the following URL in a web browser: **http://<serverIP>:<port>/ATGC/**. The administrative interface is only accessible from the server machine.

3.5 Using Apache web server

To use apache http server, you must install 2 packages and configure Web2py applications on apache configuration files with the follow commands:

$ apt-get install apache2

$ apt-get install libapache2-mod-wsgi

Download the apache configuration file from **http://atgcinta.sourceforge.net** called web2py.conf-apache.2.X (X= 2 or 4, according to apache version) and put this file in **/etc/apache2/sites-enabled**

directory with the **web2py.conf** name.

Then, open **/etc/apache2/sites-enabled/web2py.conf** file and replace:

<hostname> for the correct hostname defined in /etc/hosts file with the selected IP adress (atgc-VirtualBox in the virtual machine)

<username> for the correct username (atgc in the case of virtual machine)

<path to web2py home> for the complete path to web2py main directory (/home/atgc/web2py in the case of the virtual machine)

Put the follow two lines in **/etc/apache2/apache2.conf** file:

ServerName <hostname>

Listen 8000

Finally, you can acces using the web browser the follow ways:

http://localhost:8000/

http://hostname/

http://ip_adress/

4 Application access using the web browser

To access to the application, write this URL in the web browser: **http://localhost:8000** (other options above)

5 Application Usage

5.1 *Database creation and load ontologies (Setup menu)*

For the application usage, the first steps are: create a Chado database and load ontologies in the database. You must use the functions in the setup option of the navigation bar. When you start the application for the first time or when not exists a database, the application looks like this:

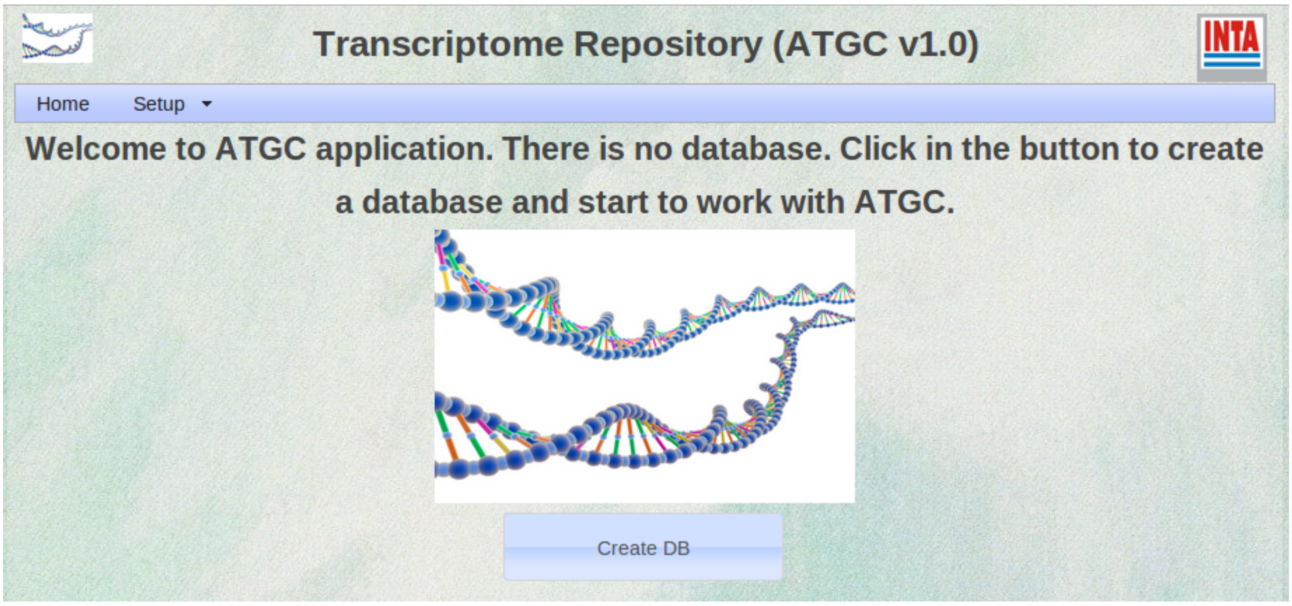

Figure 1: First step in application

5.1.1 Database creation

For database creation you only must choose a database name, and the application creates a database using a template of Chado schema and automatically load basic ontologies:

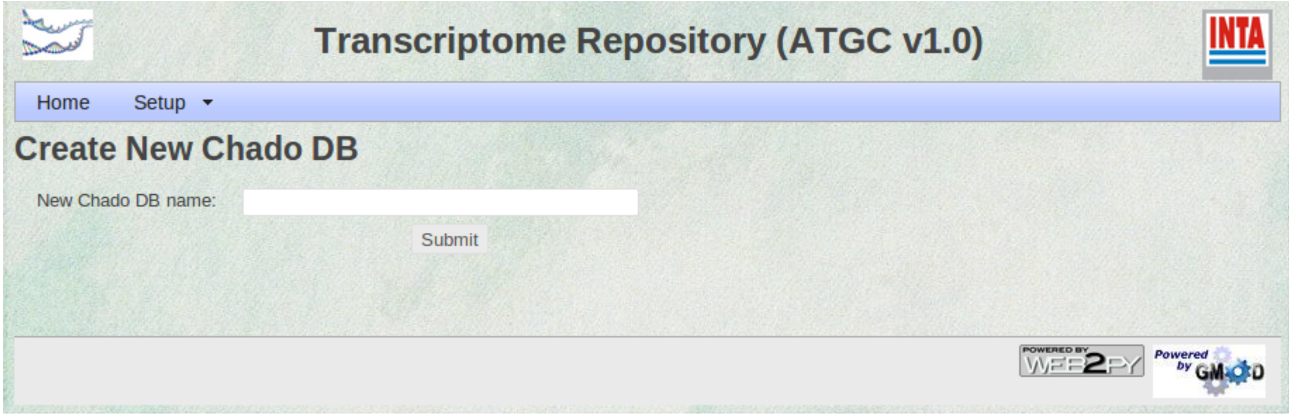

Figure 2: Database creation

5.1.2 Database selection

You can have several databases in the same instance of the application, but you must select the database with you want work.

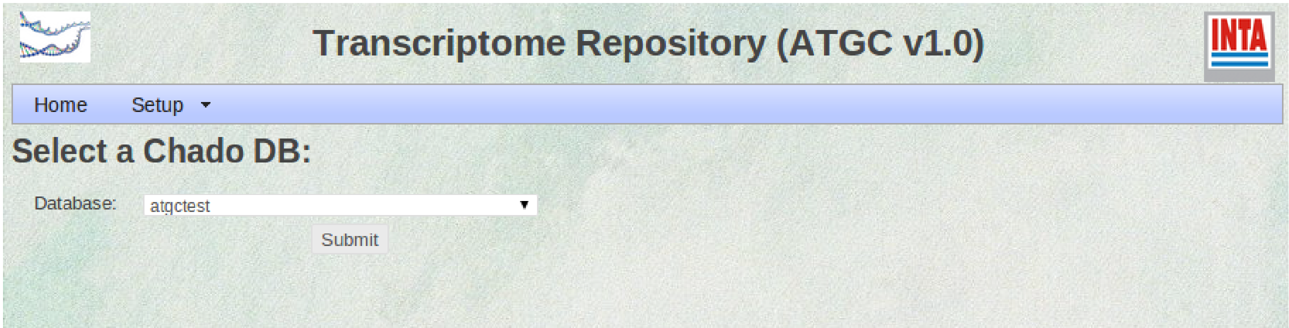

Figure 3: Database selection

5.1.3 Load Ontologies

After database creation, you must load the ontologies into the database, the main ontologies to follow with the data loading are Sequence Ontology (SO) and Gene Ontology (GO). You can load ontologies using “Load Ontology” option in Setup menu. When you load these two ontologies, you can follow loading data into the database (i.e. fasta files, GO annotations).

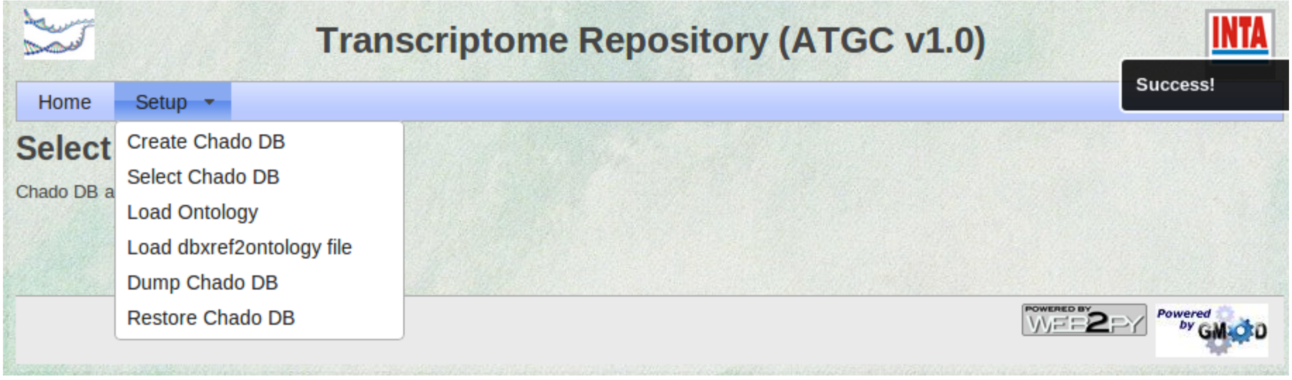

Figure 4: Load ontologies

If you use a software with a output you want load into the database using a GO ontology, you must load <software>2GO file using “Load dbxref2ontology file” option. One example of this, is InterProScan software, you can download interpro2go file from **http://geneontology.org/external2go/** and load this file to generate relationships between InterPro and GO terms into the database (after loading GO ontology).

5.1.4 Dump and Restore

The “Dump Chado DB” is used to do a backup a complete database in a “sql” compatible file format. Then is possible to use this file to restore a database to a previous state (before an error or unwanted changes) using the “Restore Chado DB” option.

5.2 Data loading

5.2.1 Create Organism

The first step then load ontologies is the creation of a organism for the database, along with this step, you must load a image to identify your organism, this image will be used in the application header (left image) and as image in the browser tab. Only is possible create one organism by database, if you need to use the application for several organisms you must create several databases (one for each organism). For this job, you don't need a new complete proccess of database creation and load ontologies, you can make a dump of the original database, create a new database and then, you can load the complete "dump" of the original database into the new database (basically creating a copy of the original database) with "Restore Chado DB".

After create an organism, you can start with loading the data, creating experiments, libraries, lines and load features from fasta files and lists files (for features without sequence). The menu looks like this:

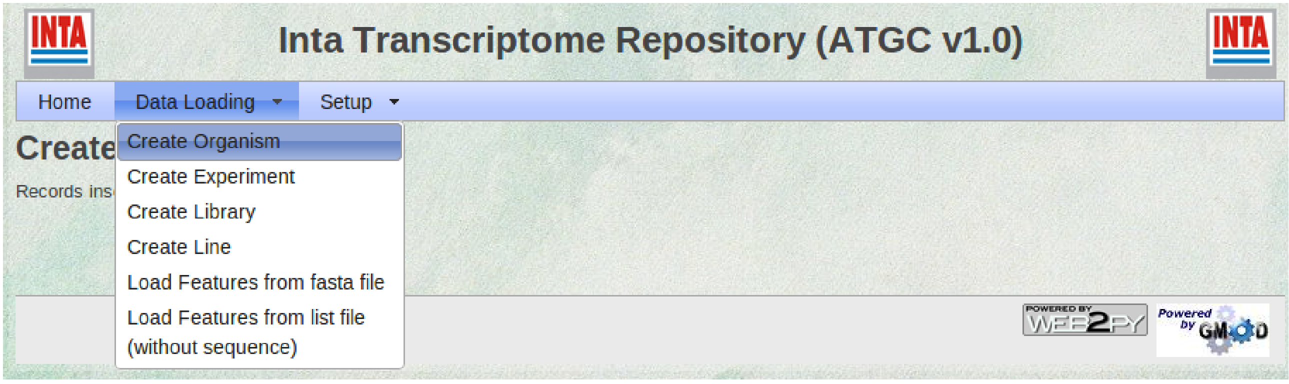

Figure 5: Data loading after create an organism

5.2.2 Load features from fasta

After creating the organism, you can load the sequences of transcripts or genes in "fasta" format, choosing the feature type for Sequence Ontology (contig for example), then all data can be loaded using this sequences as repository or reference.

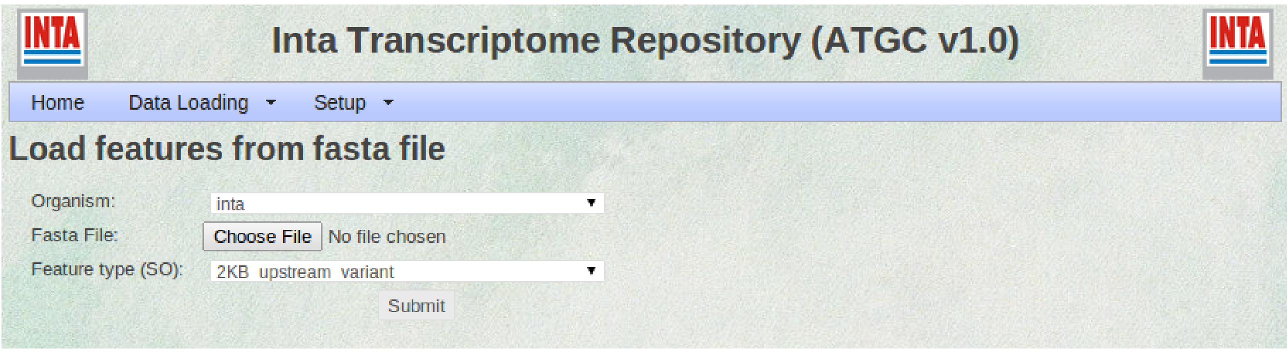

Figure 6: Load features from fasta file

Loading features in fasta format allows to add all associated information, such as, functional annotation, blast results, expression levels, markers, alleles, genotypes and relationships with other features.

5.2.3 Features → CV associations (load cvterms for features)

Associations from features to controlled vocabularies can be made using several sofware results, such as, Blast2GO (annot file), Interproscan (raw file), RFAM (gff3 file) or tab files (in general). To load Interproscan results you must load "interpro2go" file from: http://geneontology.org/external2go/, to load RFAM results you can load "rfam2go" file from the same place or "rfam2so" file developed by ATGC creators placed into: web2py/applications/ATGC/private/ontology directory. The information placed on "Description" field will be used to identificate the source of annotations in feature detail pages.

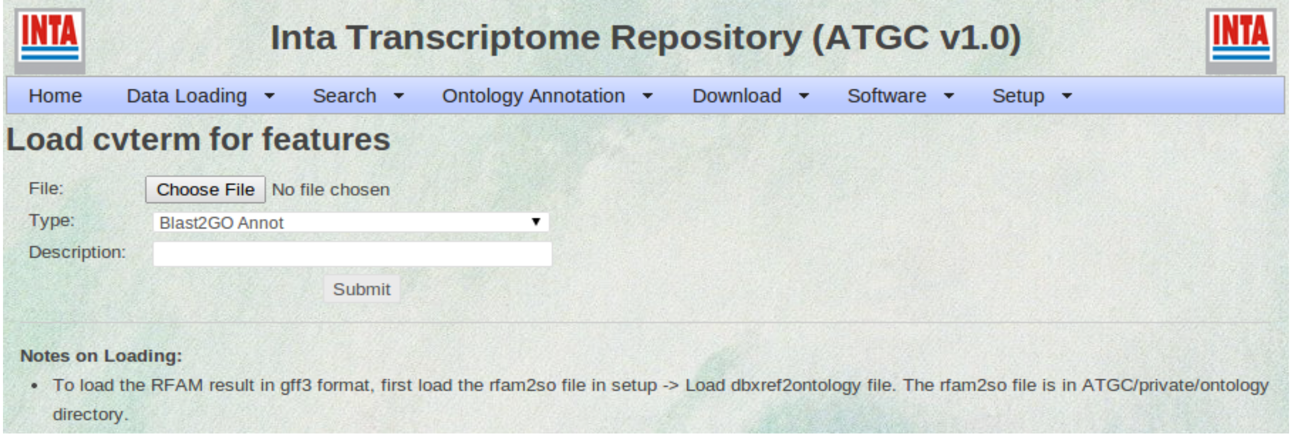

Figure 7: Feature → CV associations

5.2.4 Blast run results (XML Files)

In the same way that CV association, is possible to load the results of Blast alignments against a database using xml as output type.

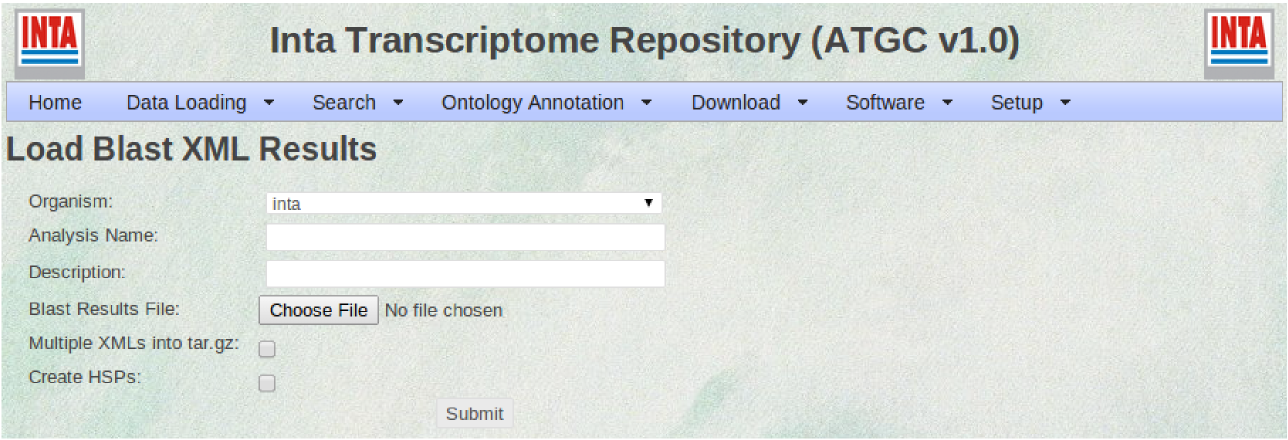

Figure 8: Load Blast results

5.2.5 Genotypes (lines), markers and alleles

You can load a set of markers and genotypes defined by alleles of this markers, to make this, you must load first a genotypes and then the markers and alleles.

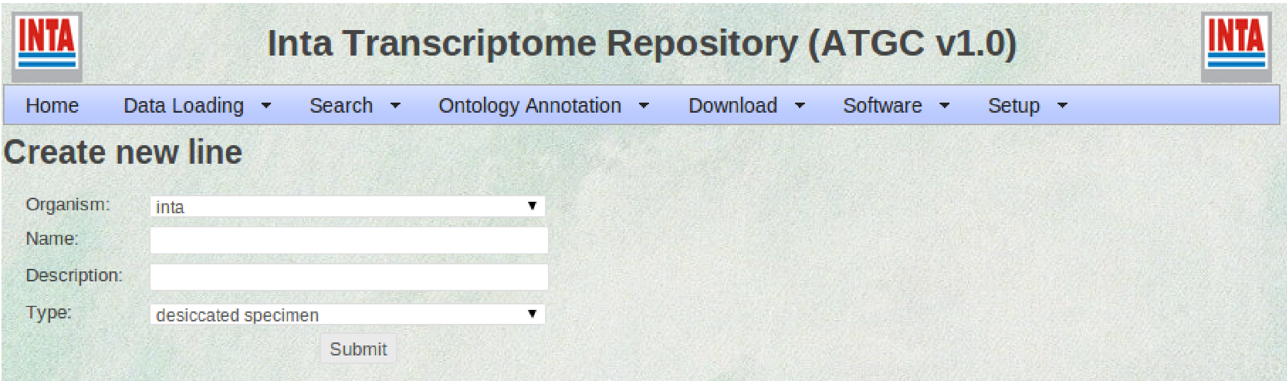

Figure 9: Create new line

Is possible to describe the characteristics of the genotypes using terms of controlled vocabularies, in the tab of Lines → CV associations, first choosing the CV (for example INTA_CV or any ontology previously loaded) and then adding a characteristic and value for this line.

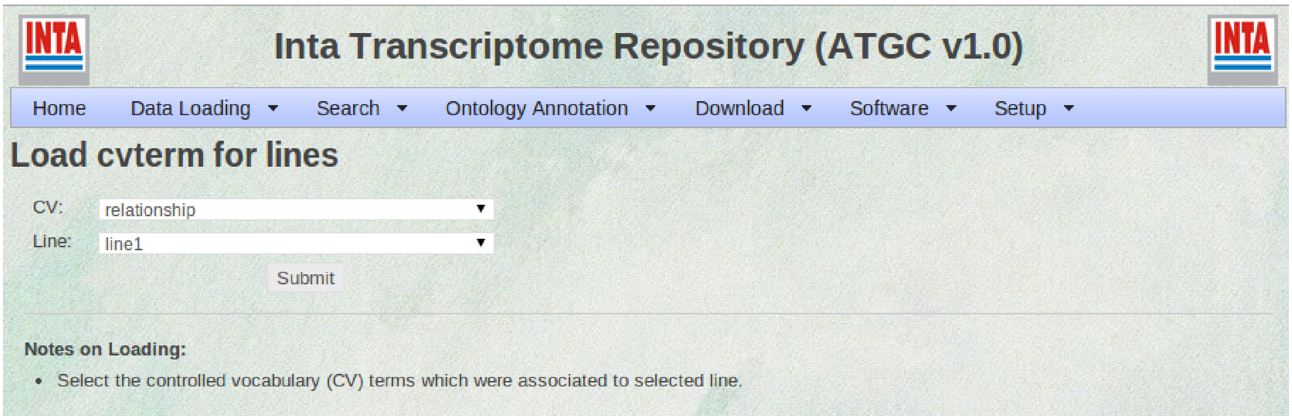

Figure 10: Lines → CV associations

For example, setting the characteristic “Fertility” on “Yes”

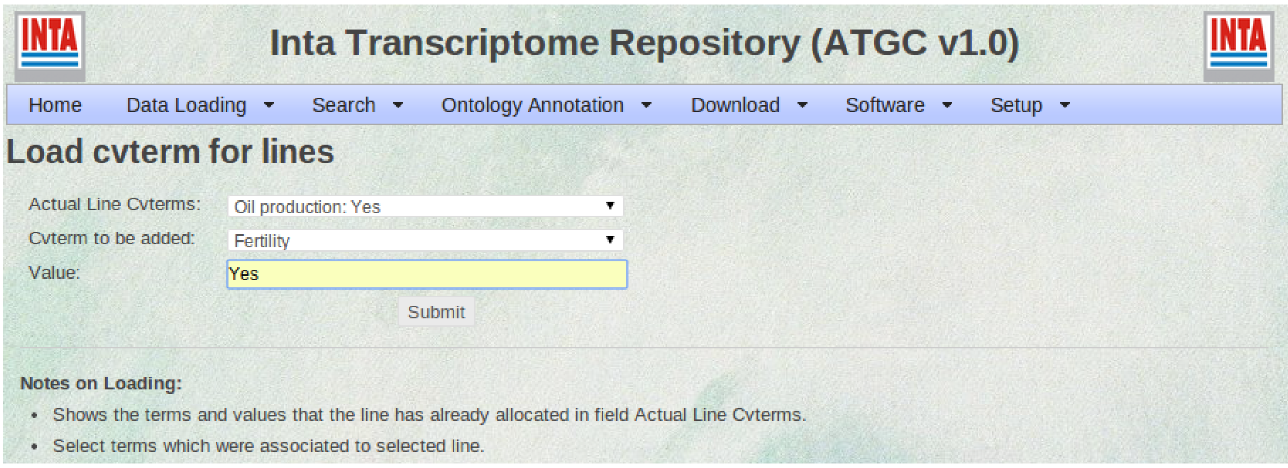

Figure 11: Lines → CV associations

To load markers you can use Load Markers section, given the markers infomation on several file types, such as: VCF, CSV or specific formats of some sofwares. If you want to create a relation between markers and genotype, you must to select the option “Allele creation and line association” and select the genotype name.

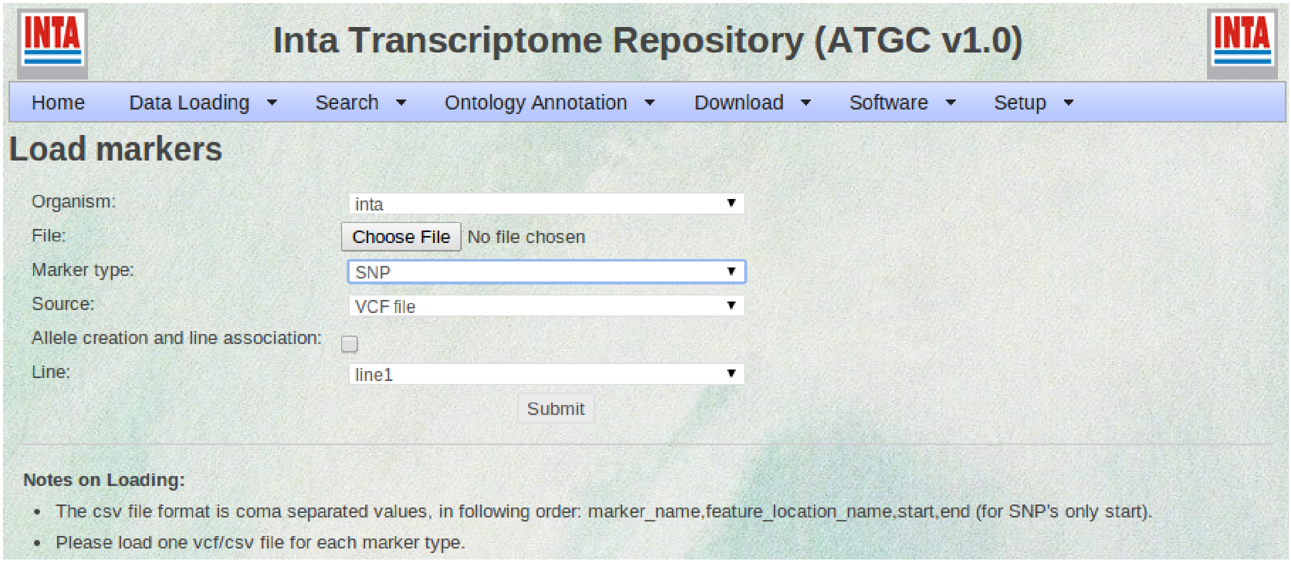

Figure 12: Load markers

In the same way, if you want create an association of marker with other line, you can use the window: Markers → Line associations, from a csv file to describe the allele of each marker in the new line.

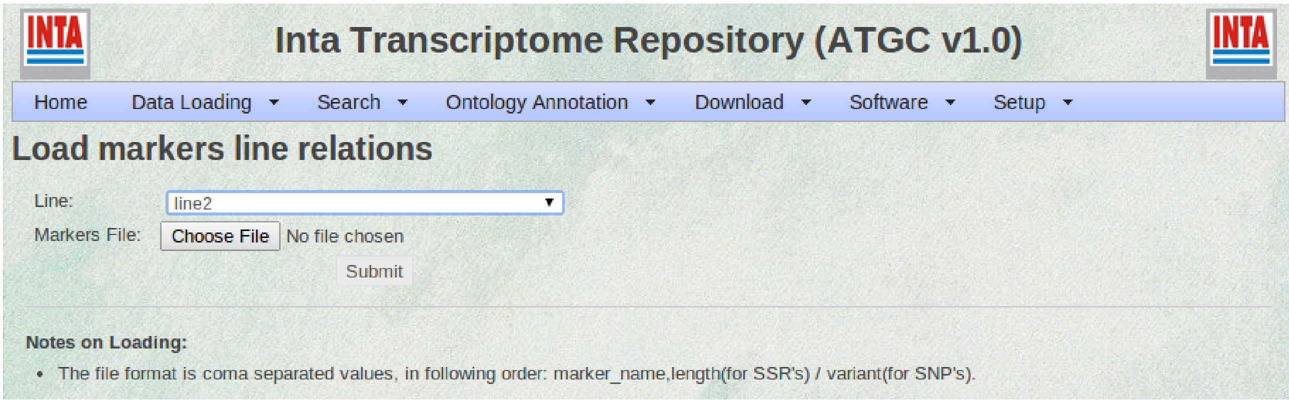

Figure 13: Markers → Line associations

5.2.6 Load expression information

To load expression information in the database, you first create a estructure of experiments (with your characteristics, any treatment in the assay is a different experiment, for example, if you have two treatments, control and treated, you must to create two experiments) and libraries (like biological or technical replicates) and finally you can use the “Feature → Library associations” window to load expression or other measure variables to any feature (contigs for example).

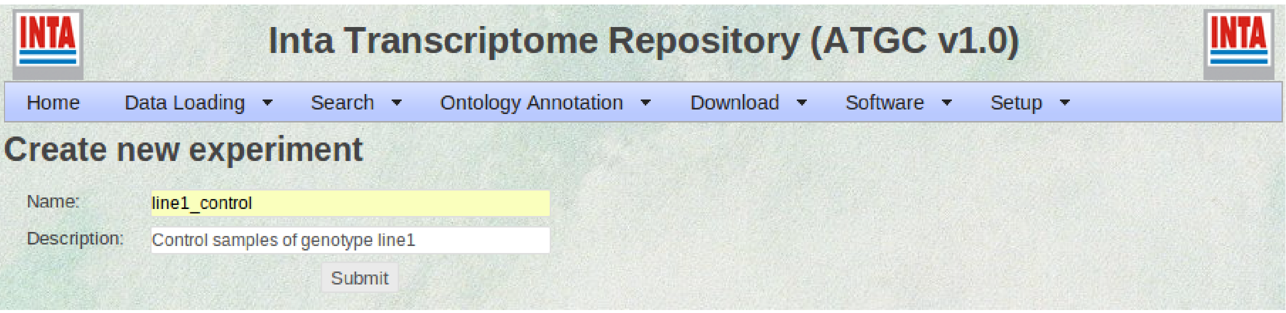

Figure 14: Create experiment

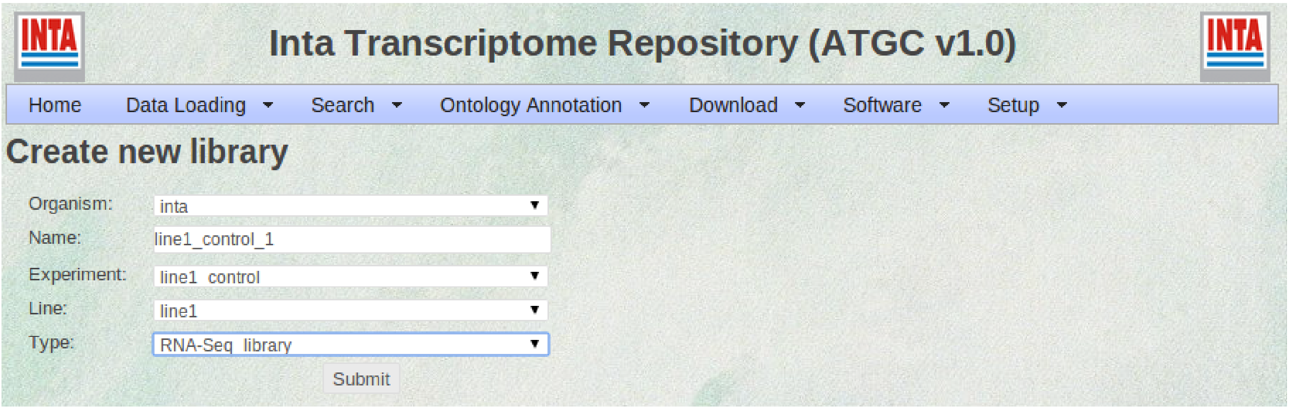

Figure 15: Create library associatted to a line and experiment

Is very important to add information to fully describe the experiments, then this information will be used to create dynamic expression graphics in feature detail pages.

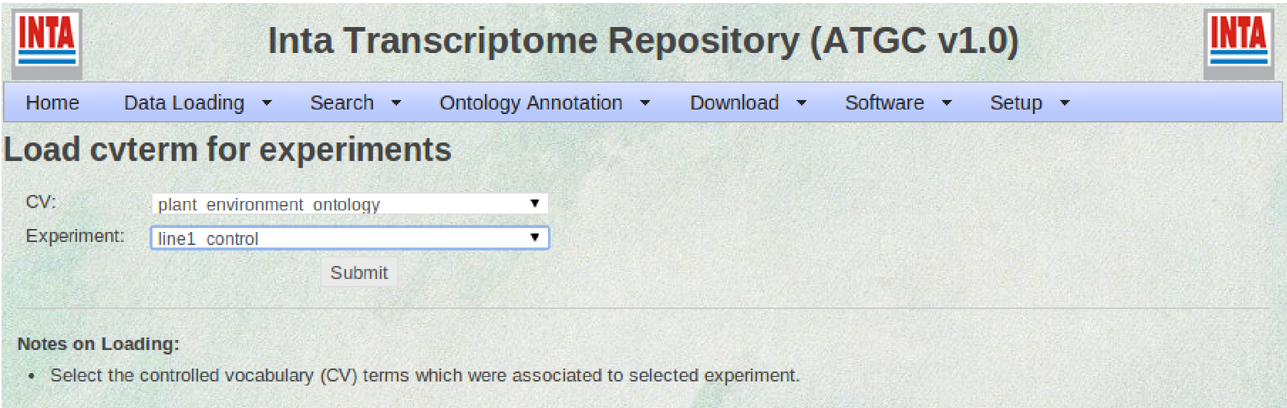

Figure 16: Experiments → CV associations

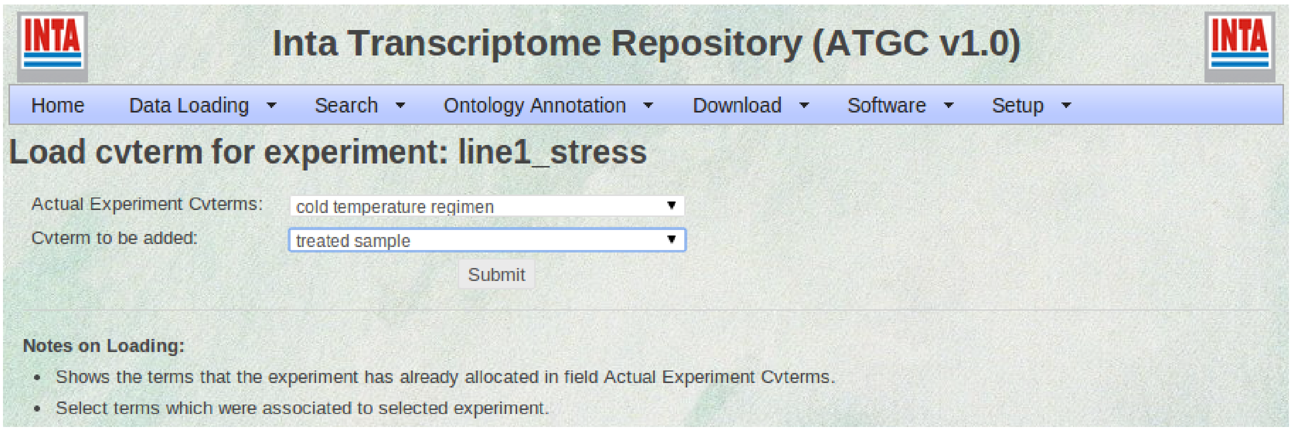

Figure 17: Experiments → CV associations (second part)

With the structure of experiments and libraries created, you can load to the database the information of features related with each library, for example, in the Figure 18 the file contains values of RPKM counts of expression.

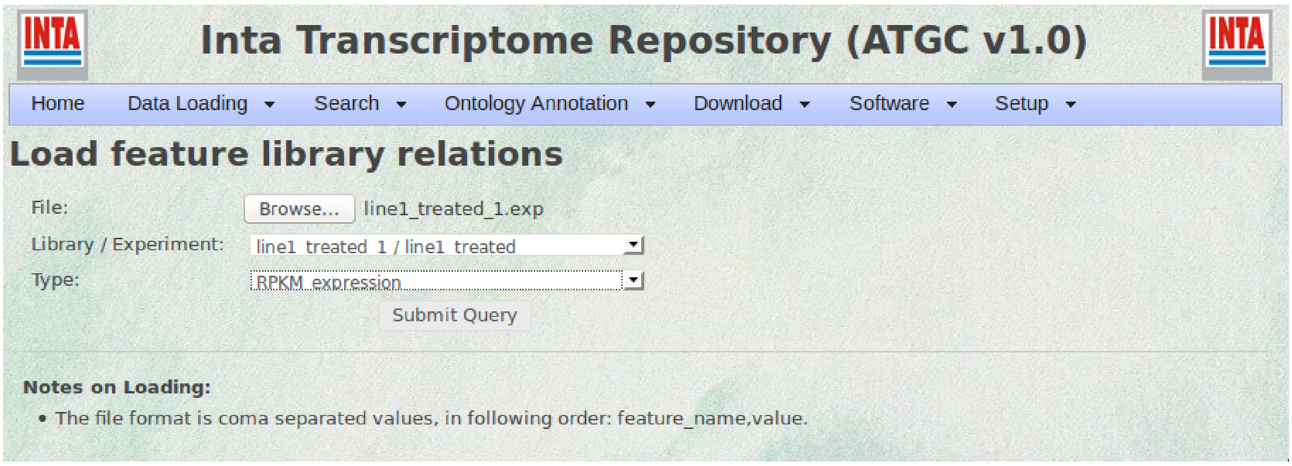

Figure 18: Feature → Library associations

5.2.7 Feature relationships

You can load relationships between features, for example, if you predict clusters of contigs that possible come from the same gene, you can load this information using the option of Figure 19, you must define the type of the relationship using ontology terms and load previously contigs and genes as features.

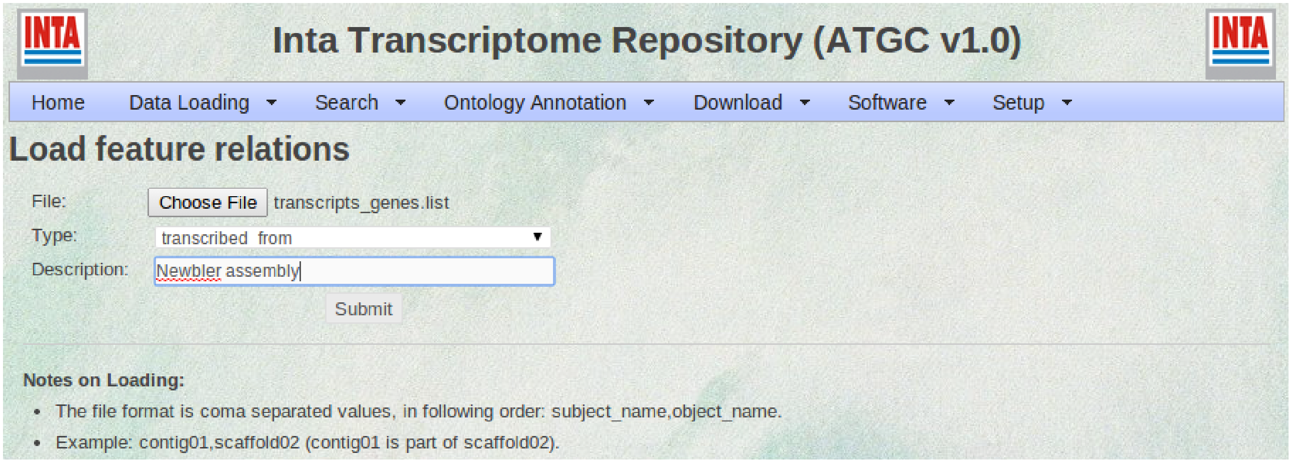

Figure 19: Load relationships between features

Other posibility to load feature relationships is using gff3 files, and you can choose the type of parental relationship between features (for example, relation between exon and transcript), in this case, is only necesary have loaded the references of the gff3 file as features in the database.

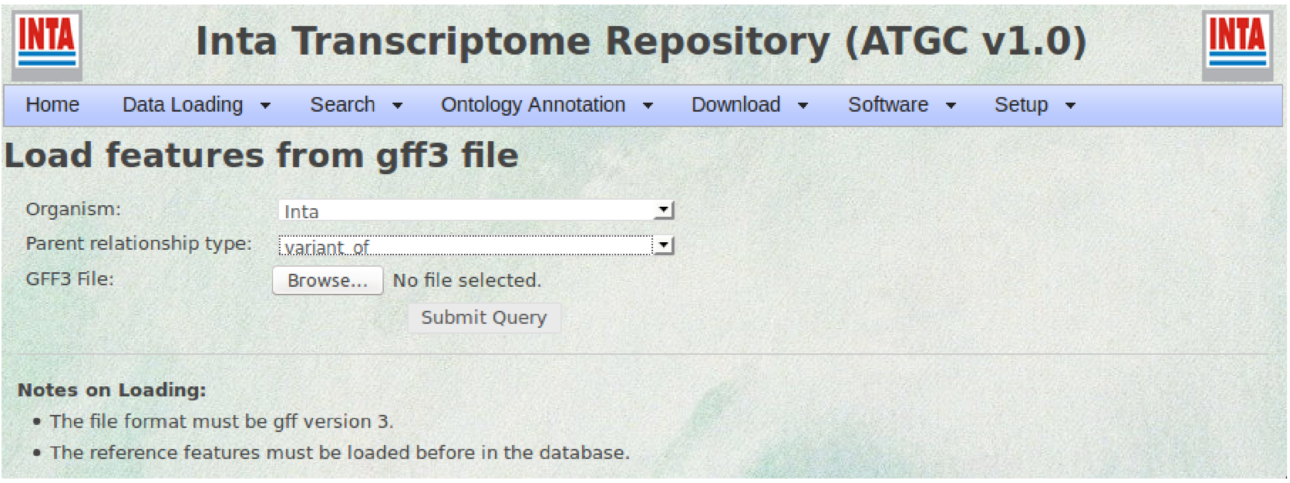

Figure 20: Load features relationships from gff3 file

5.3 Sequence search

5.3.1 Search features by name

Using the application, you can explore all information related to the features from different ways, one of this ways, is search features from the name of the feature, or an expression related with the name (using % character as wildcard)

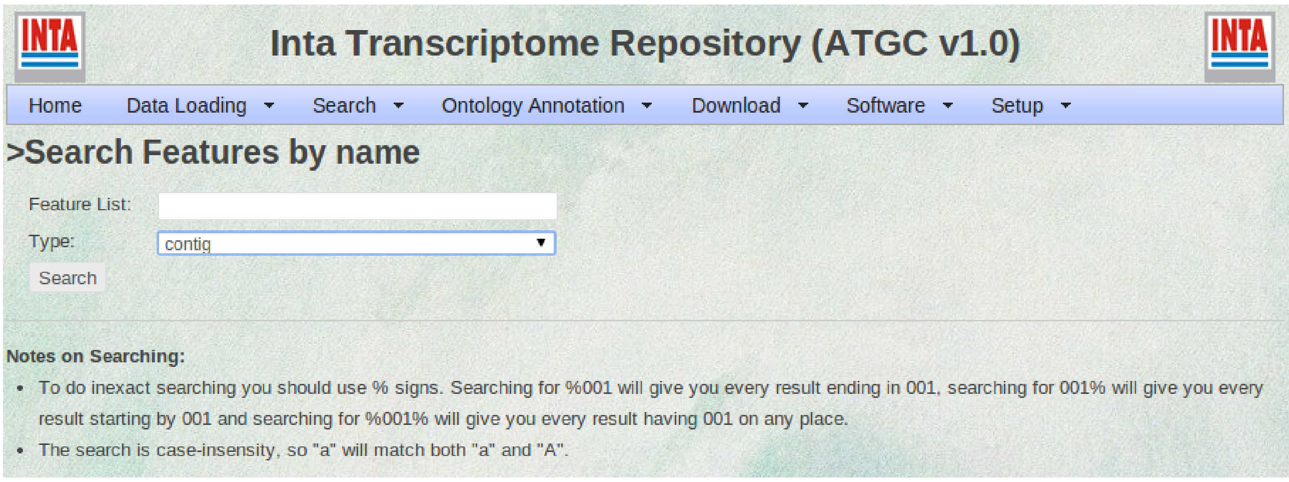

Figure 21: Search by name

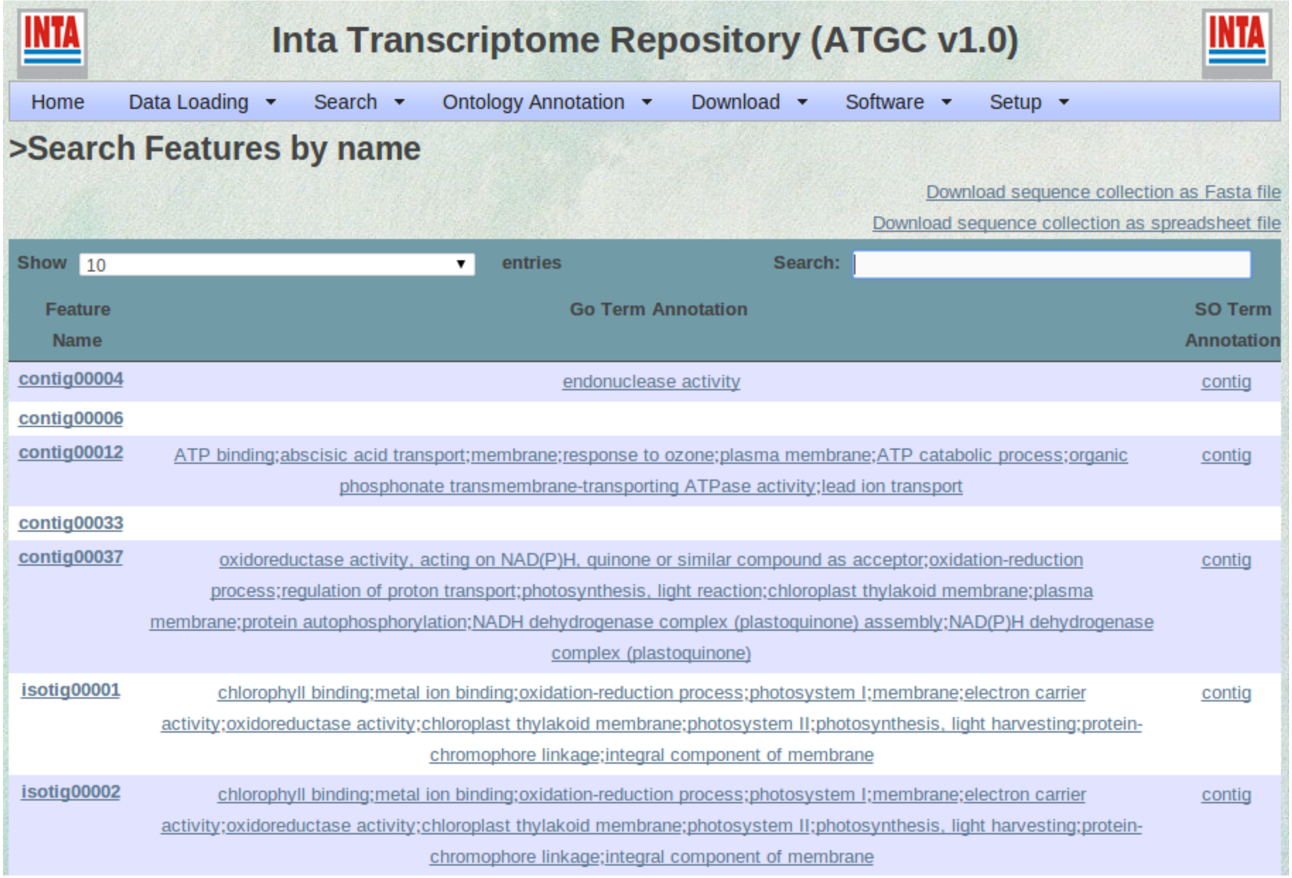

Figure 22: Search by name result

5.3.2 Search features by list of names

If you have a list of features of interest for any reason, you can search information for these features using this option

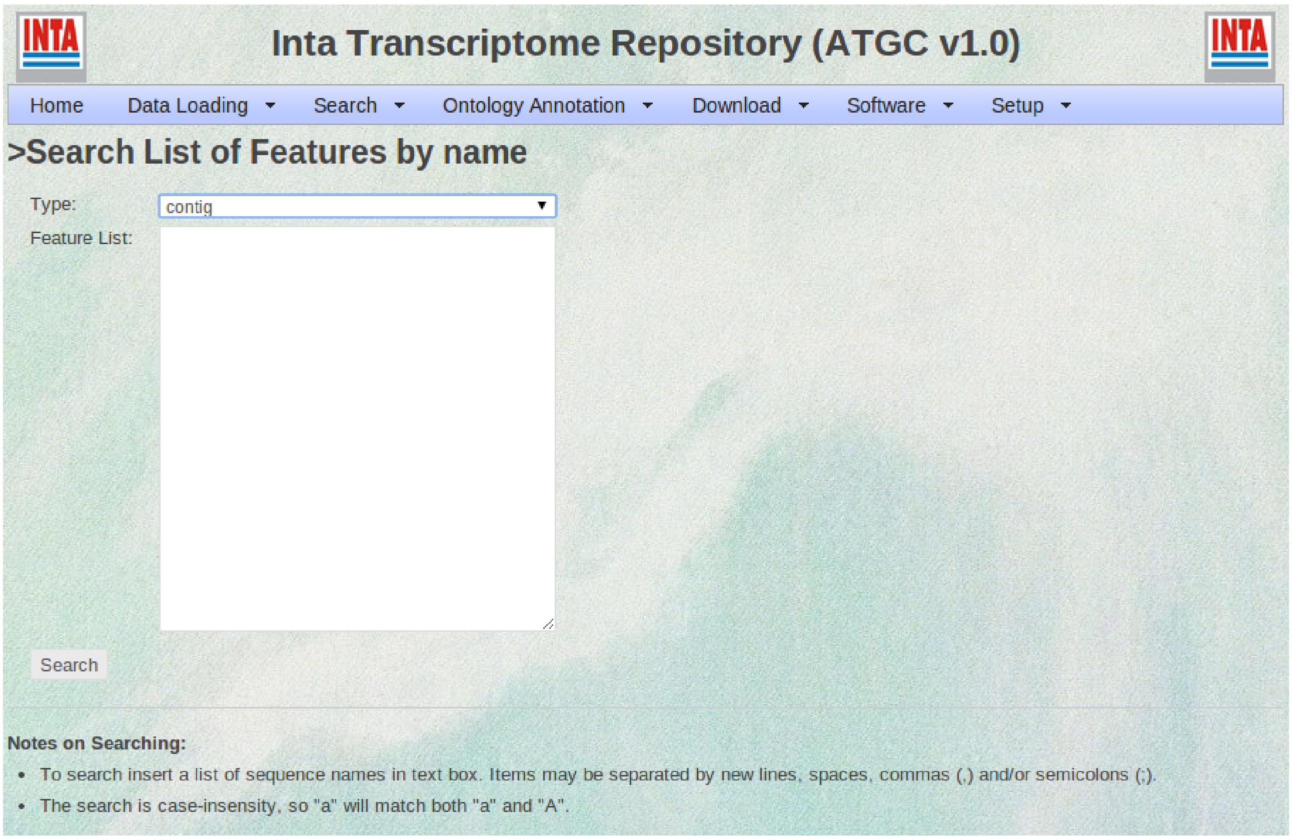

Figure 23: Search list of features by name

5.3.3 Search features by ontology term name or accession

In the other way, you can search features by functional annotation, using ontology term name or accession, and then obtain the list of features annotated with this ontology term.

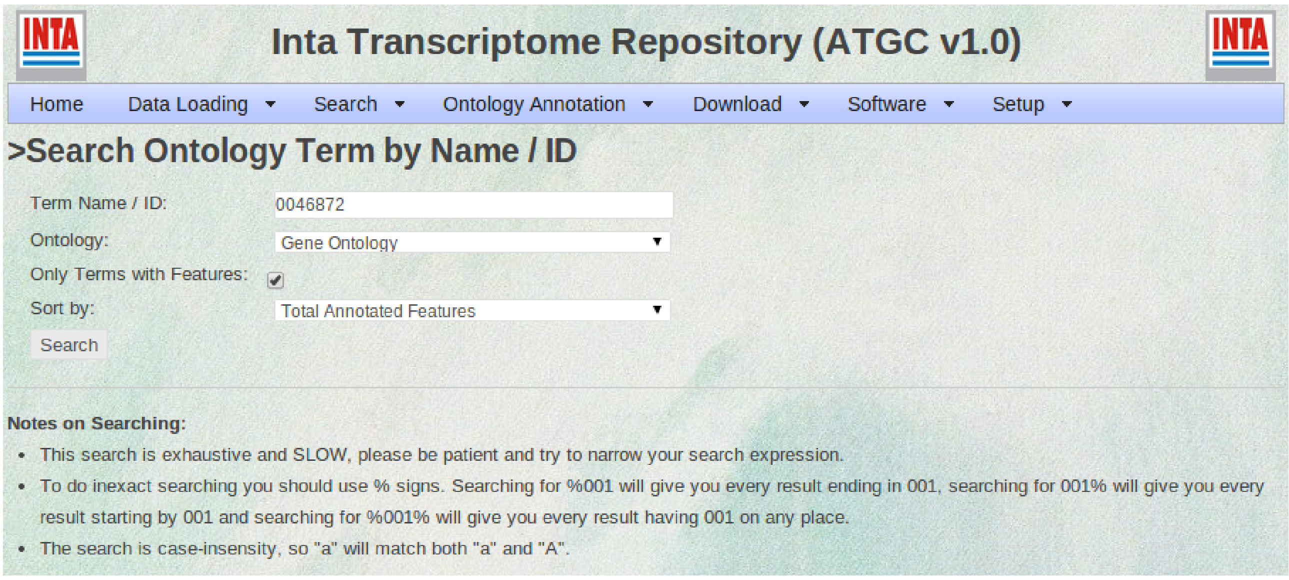

Figure 24: Search features by ontology annotation

The results of these searches can be see on two formats, the list of features annotated (direct and indirect) called term list view or a summary called term list view, with only the amount of features of each condition.

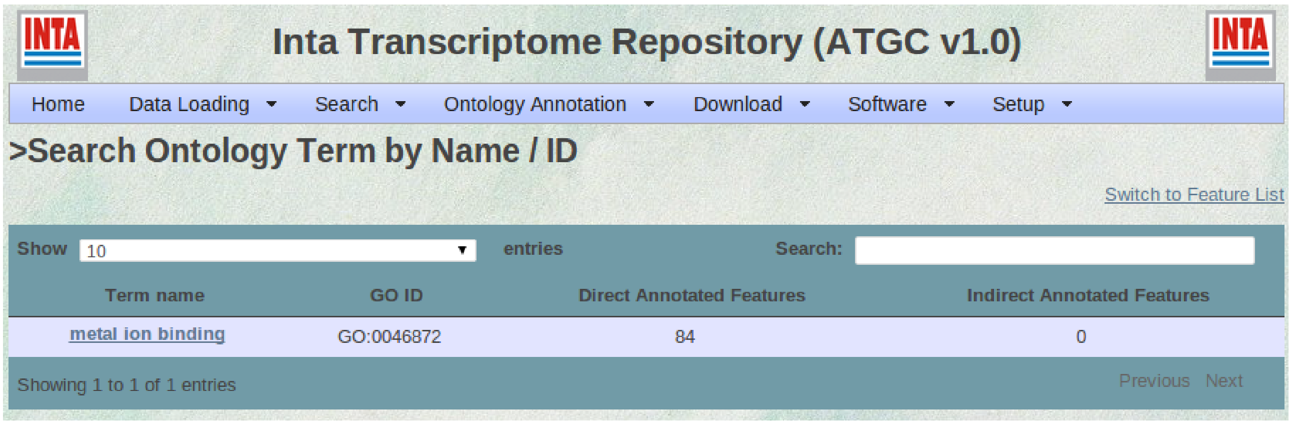

Figure 25: Search by ontology, summary result

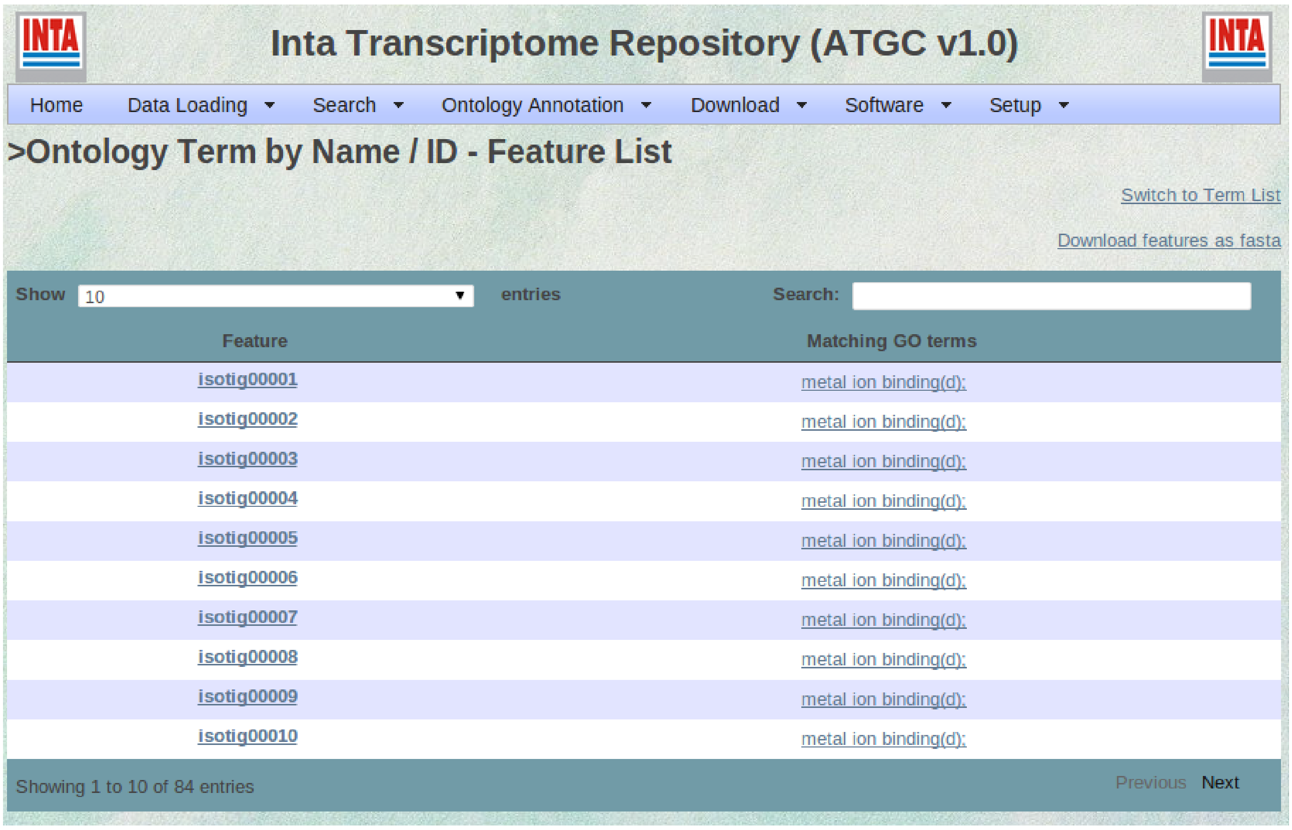

Figure 26: Search by ontology, feature list result

5.3.4 Search by Blast matches

The last way, is search through the headers or descriptions of blast results for each transcript, such as a more general and nonspecific form to relate features with functional annotation or similar sequences.

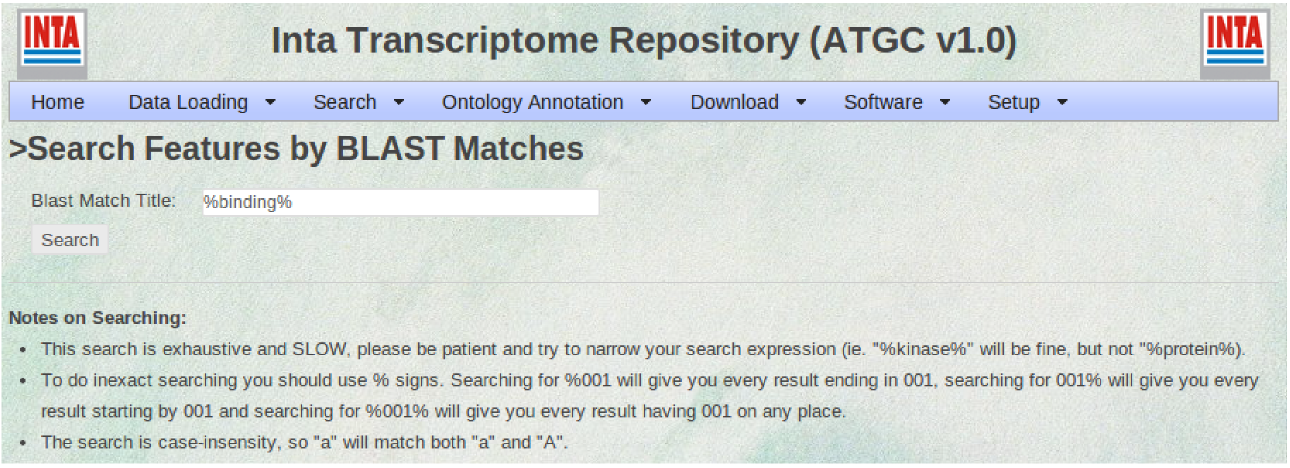

Figure 27: Search features by Blast matches

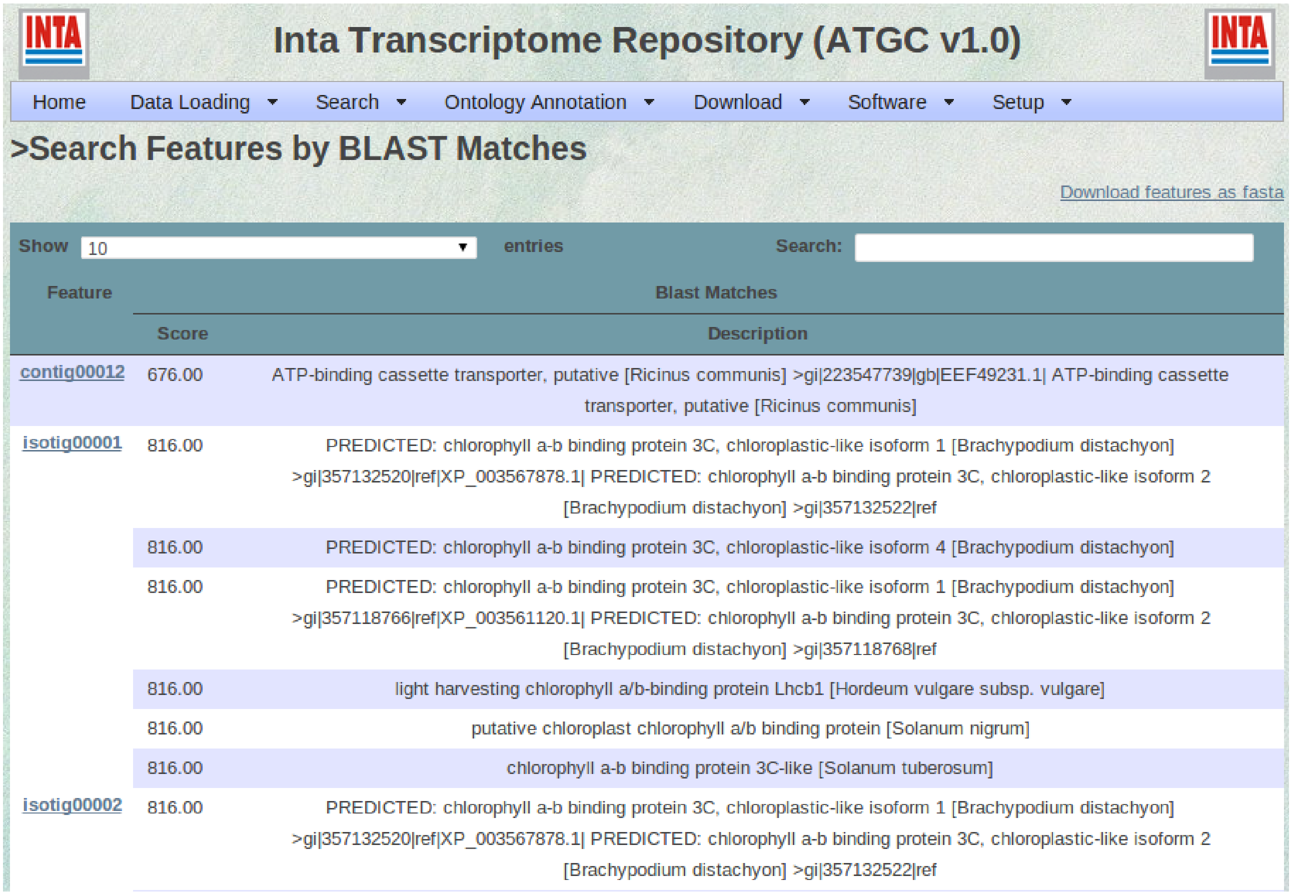

Figure 28: Search by Blast matches results

5.4 Ontology exploration

You can explore the data across the ontology using the graph structure of terms and connections, showing a pie chart with the distibution of annotated features of each ontology term and a dropdown menu to move along the graph.

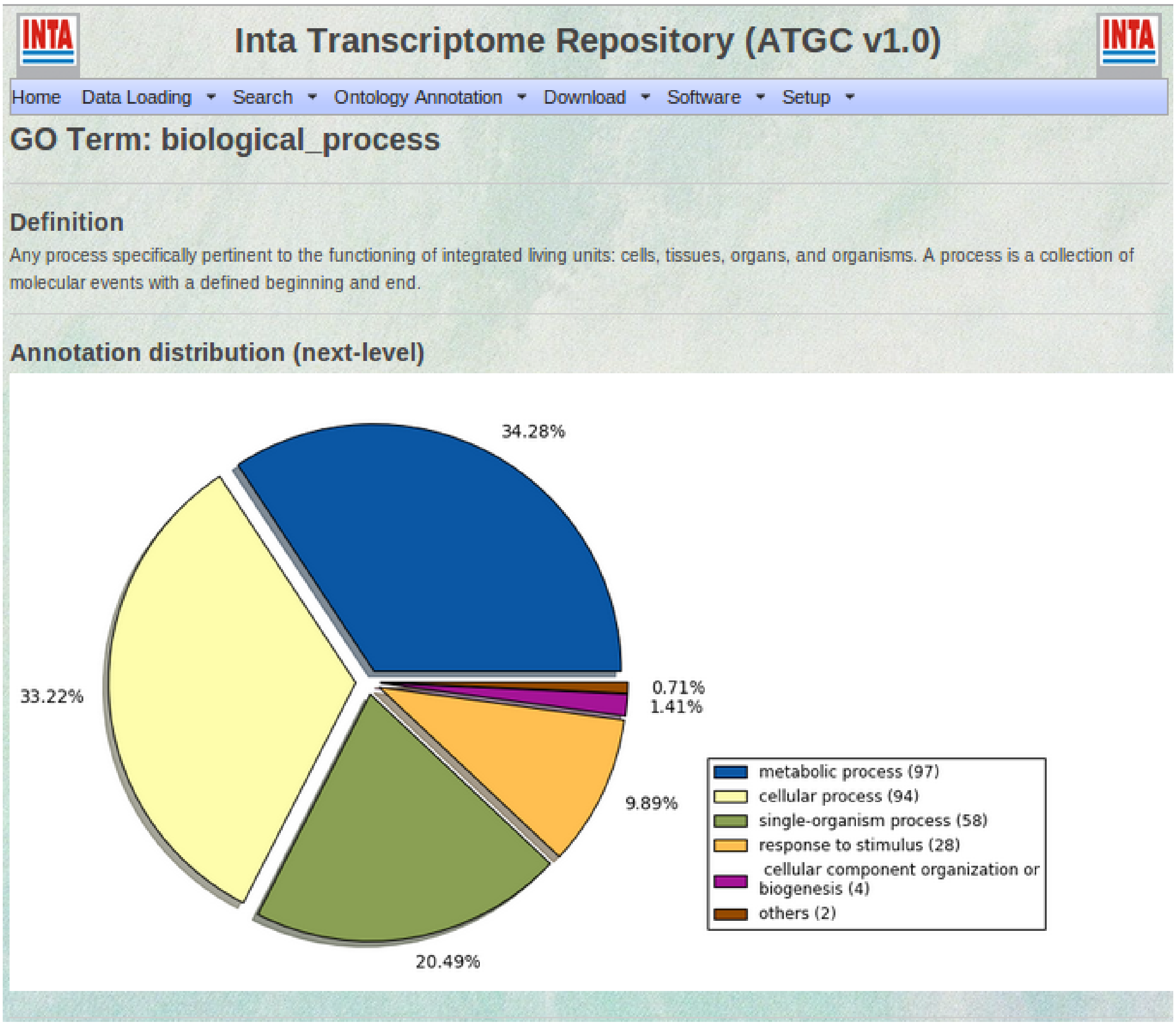

Figure 29: Pie chart: Biological process

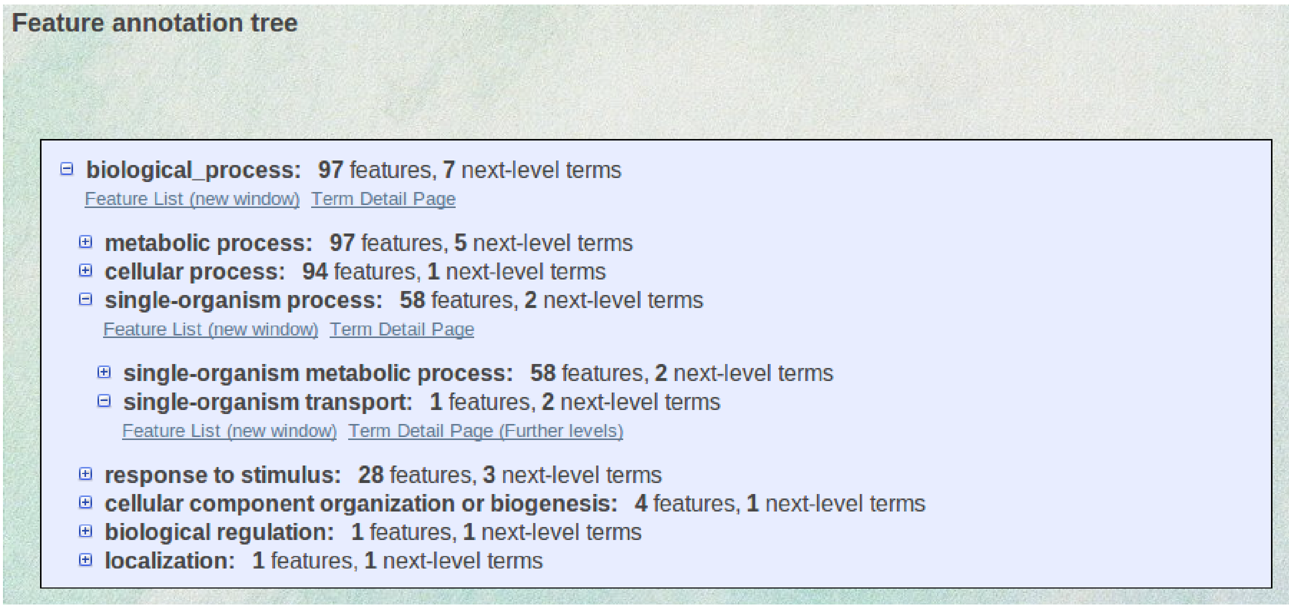

Figure 30: Feature annotation tree (Dropdown menu)

5.5 Download

Using the Download section, is possible to obtain sequences and annotations from database to text files to make other analysis, such as, functional enrichment. You can select the sequence type and download directly the file in fasta format or download the complete set of functional annotations in tabular format.

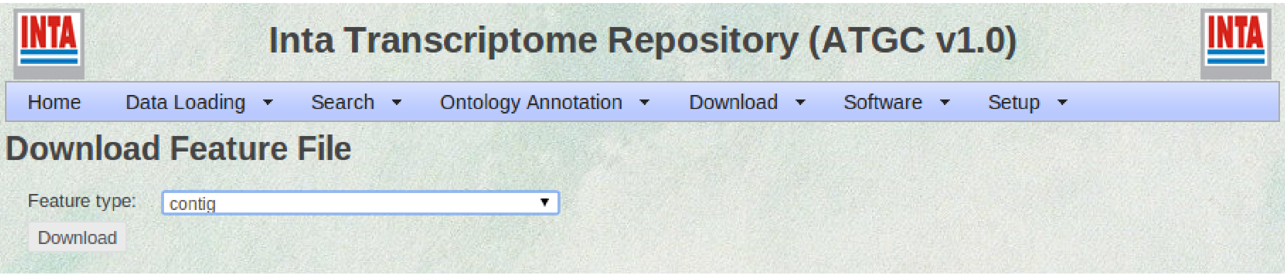

Figure 31: Download feature file

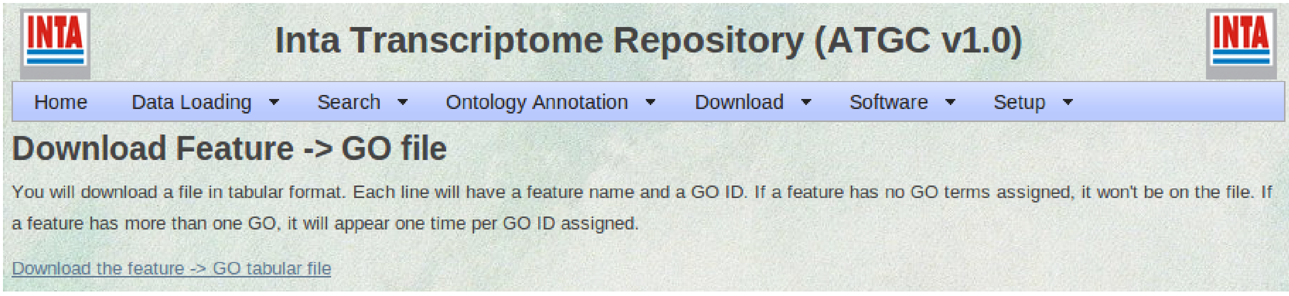

Figure 32: Download annotations

5.6 Software

Using the application is possible to make Blast alignments from sequences stored in the database as subject and external sequences as query.

5.6.1 Blast

You can make blast aligments of query sequences using the sequences stored in the database as subject. The first step to make this alignments is to create a database choosing the sequences of interest from the database. For example in the Figure 33 the user is creating a Blast database called contigs that contain the sequences of all contigs stored in the database.

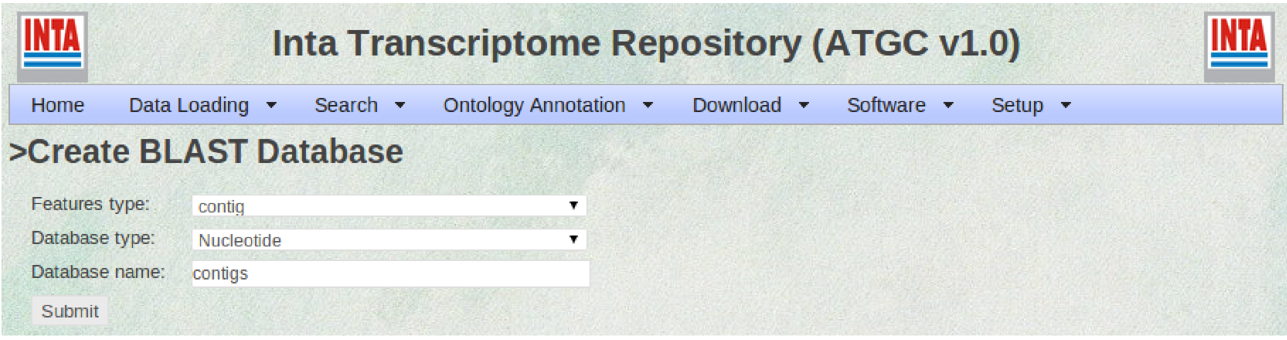

Figure 33: Create Blast database

With the database created, you can make the alignment using the option Software → BLAST → Run BLAST, in this place is possible to choose the type of Blast alignment among the options: BlastN, TblastN or TblastX. The query sequence or sequences can be inserted directly in the text box or using a text file.

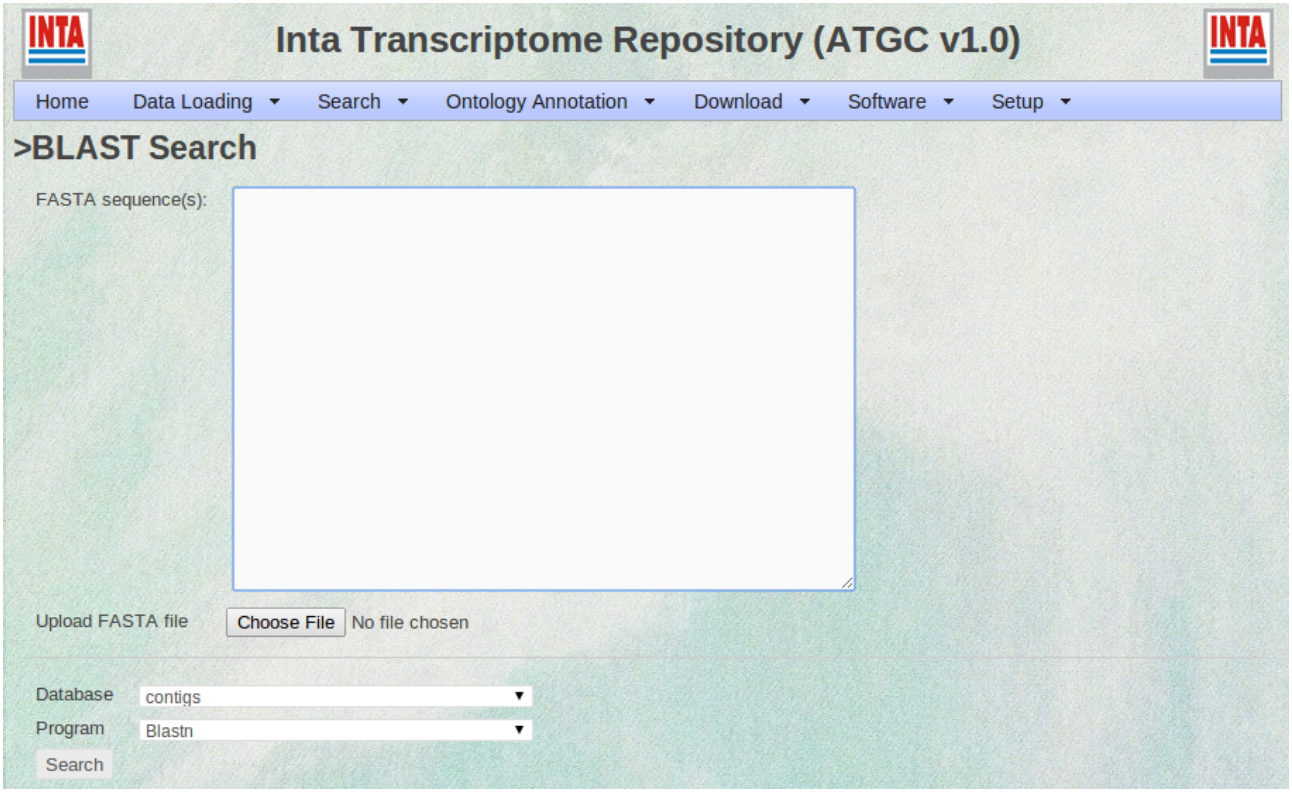

Figure 34: Run Blast alignments

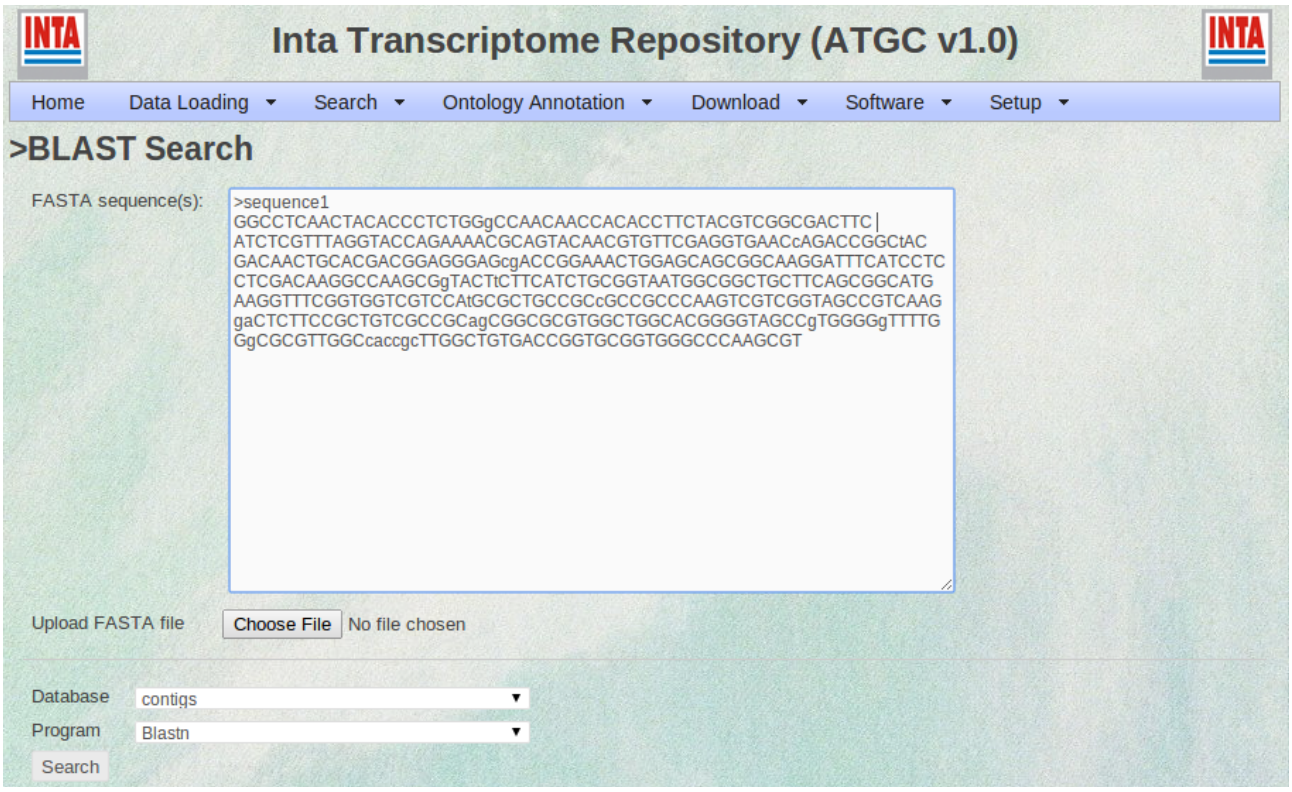

Figure 35: Example of Blast alignment

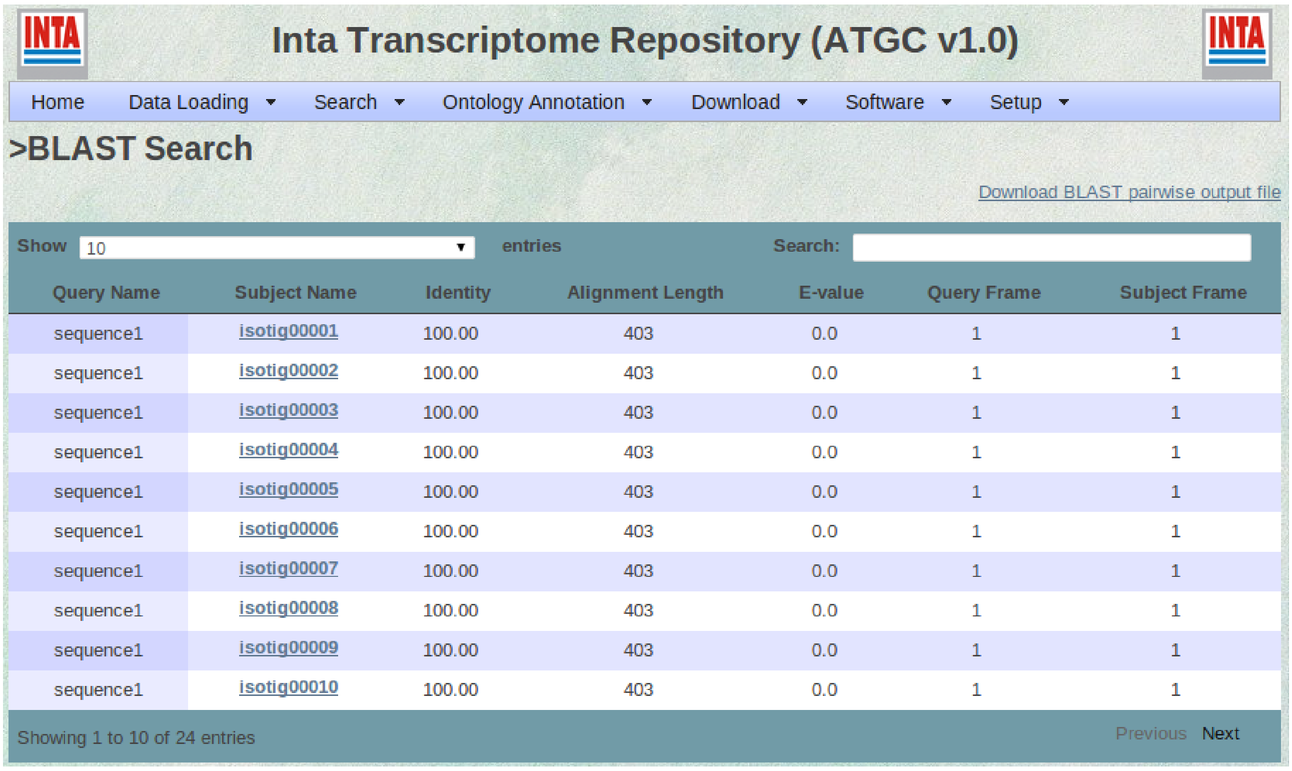

Query Name	Subject Name	Identity	Alignment Length	E-value	Query Frame	Subject Frame
sequence1	isotig00001	100.00	403	0.0	1	1
sequence1	isotig00002	100.00	403	0.0	1	1
sequence1	isotig00003	100.00	403	0.0	1	1
sequence1	isotig00004	100.00	403	0.0	1	1
sequence1	isotig00005	100.00	403	0.0	1	1
sequence1	isotig00006	100.00	403	0.0	1	1
sequence1	isotig00007	100.00	403	0.0	1	1
sequence1	isotig00008	100.00	403	0.0	1	1
sequence1	isotig00009	100.00	403	0.0	1	1
sequence1	isotig00010	100.00	403	0.0	1	1

Figure 36: Example of Blast result

5.7 Modify and delete

You can modify and remove data stored in the database using the section “Modify and Delete”, having two options to select the work entry: Using the entry name or selecting the entry from a complete list of features.

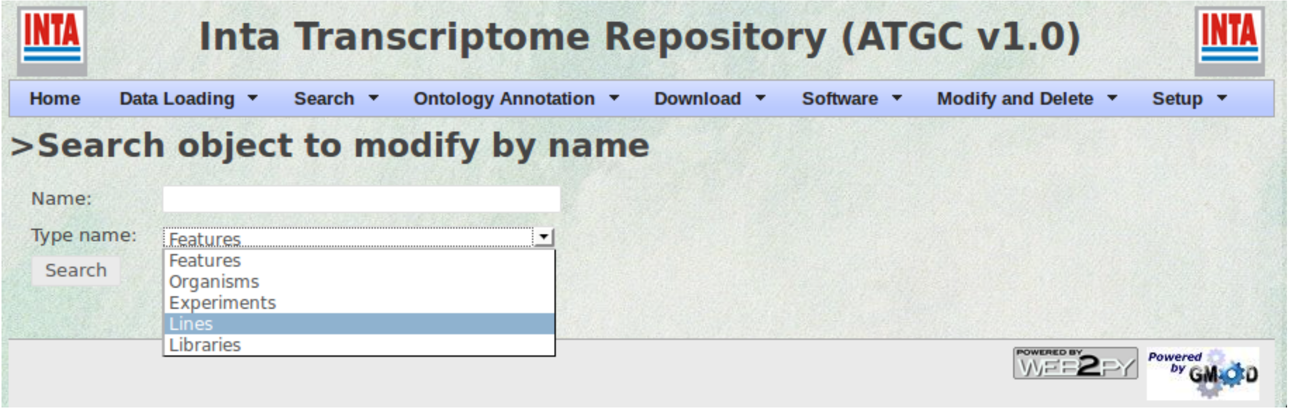

Figure 37: Search object to modify

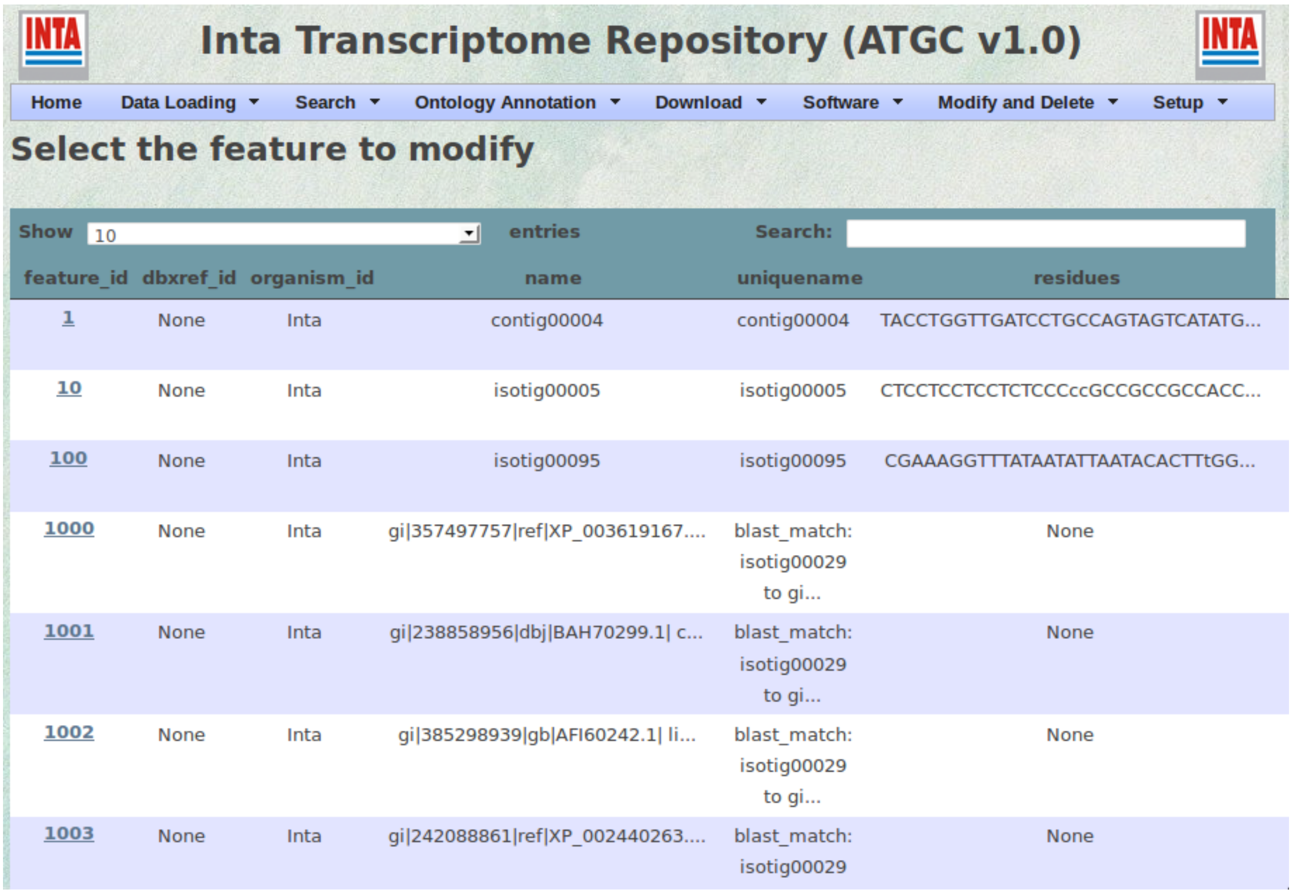

Figure 38: Table to select object to modify

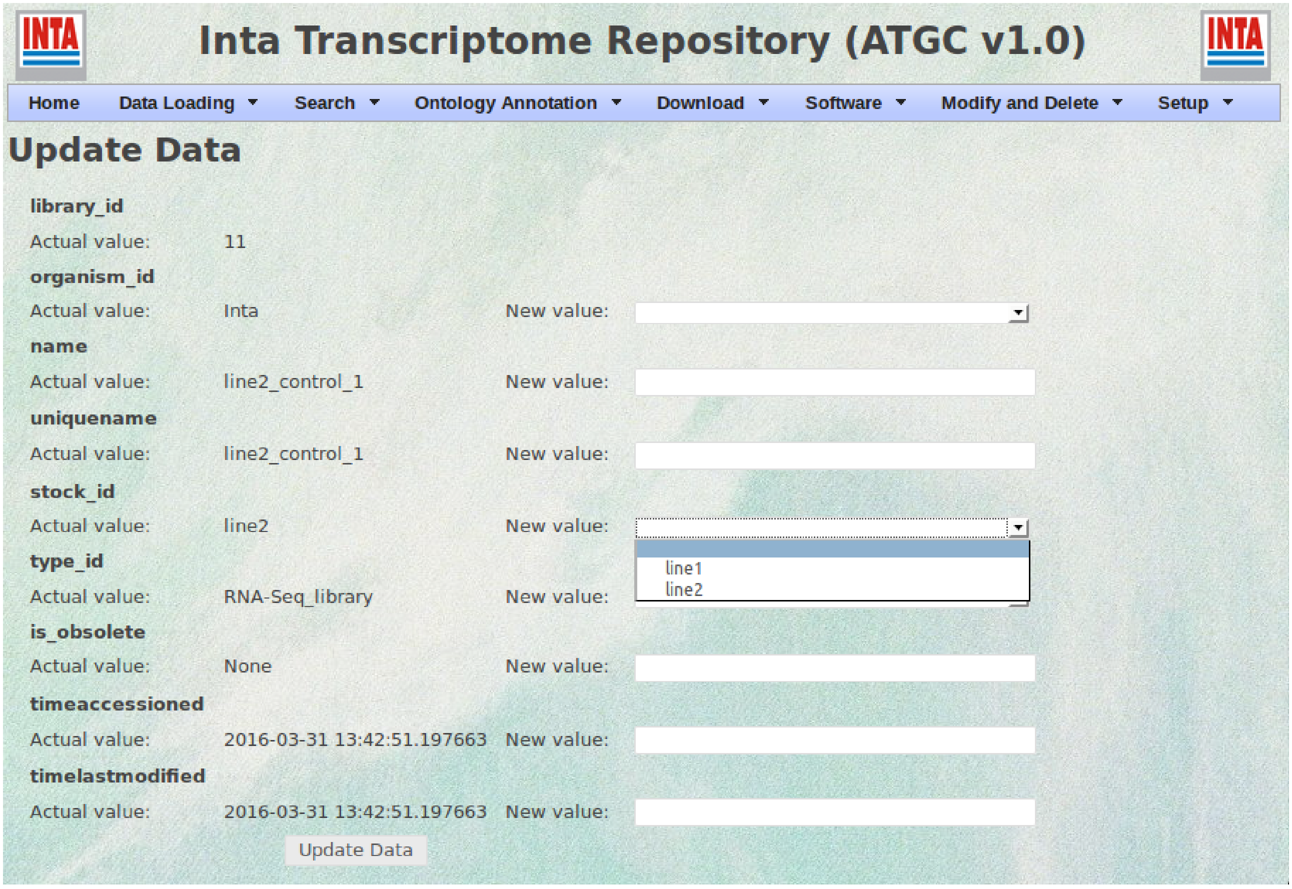

Figure 39: Update form to modify values

6 Share information

To share information, you can use a Web2py internal web server or install and configure apache web server, in any way you can choose that part of menu bar is visible to other people ass follows:

Go to the follow direction in the web browser and enter the password choosen when you run Web2py (Web2py administrative interface, only available whit the Web2py web server):

localhost:8000/admin

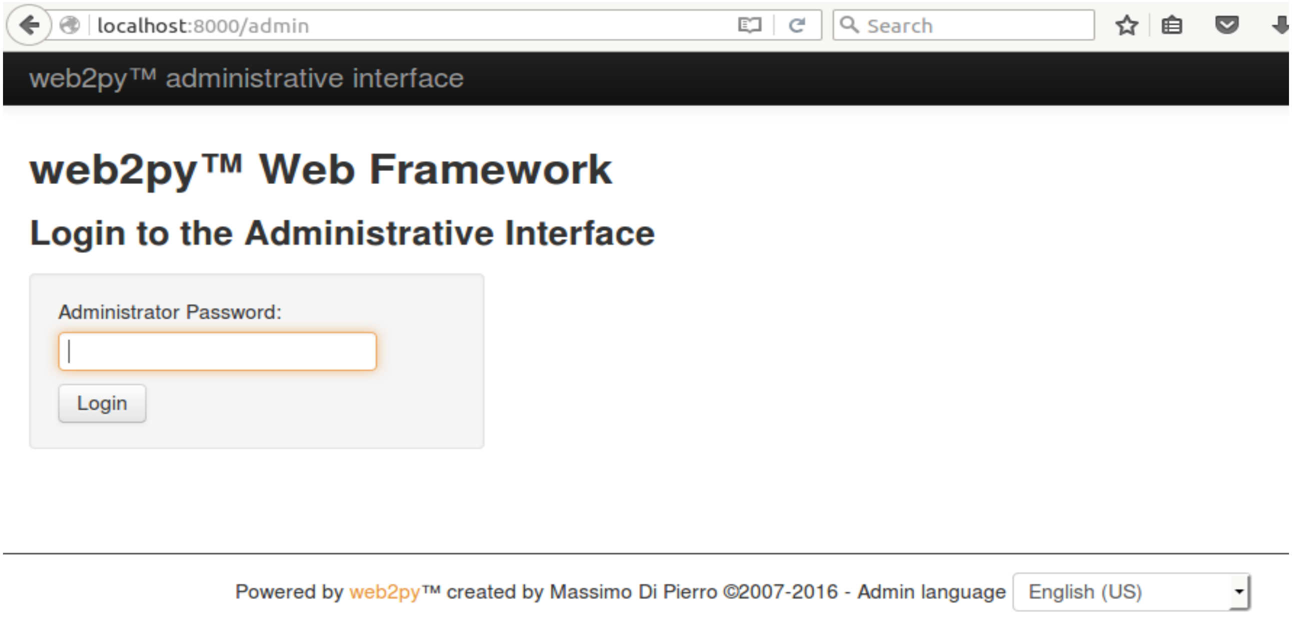

Figure 40: Web2py administration interface: write a password

In the first page, you must click on “Manage” and choose “Edit” option

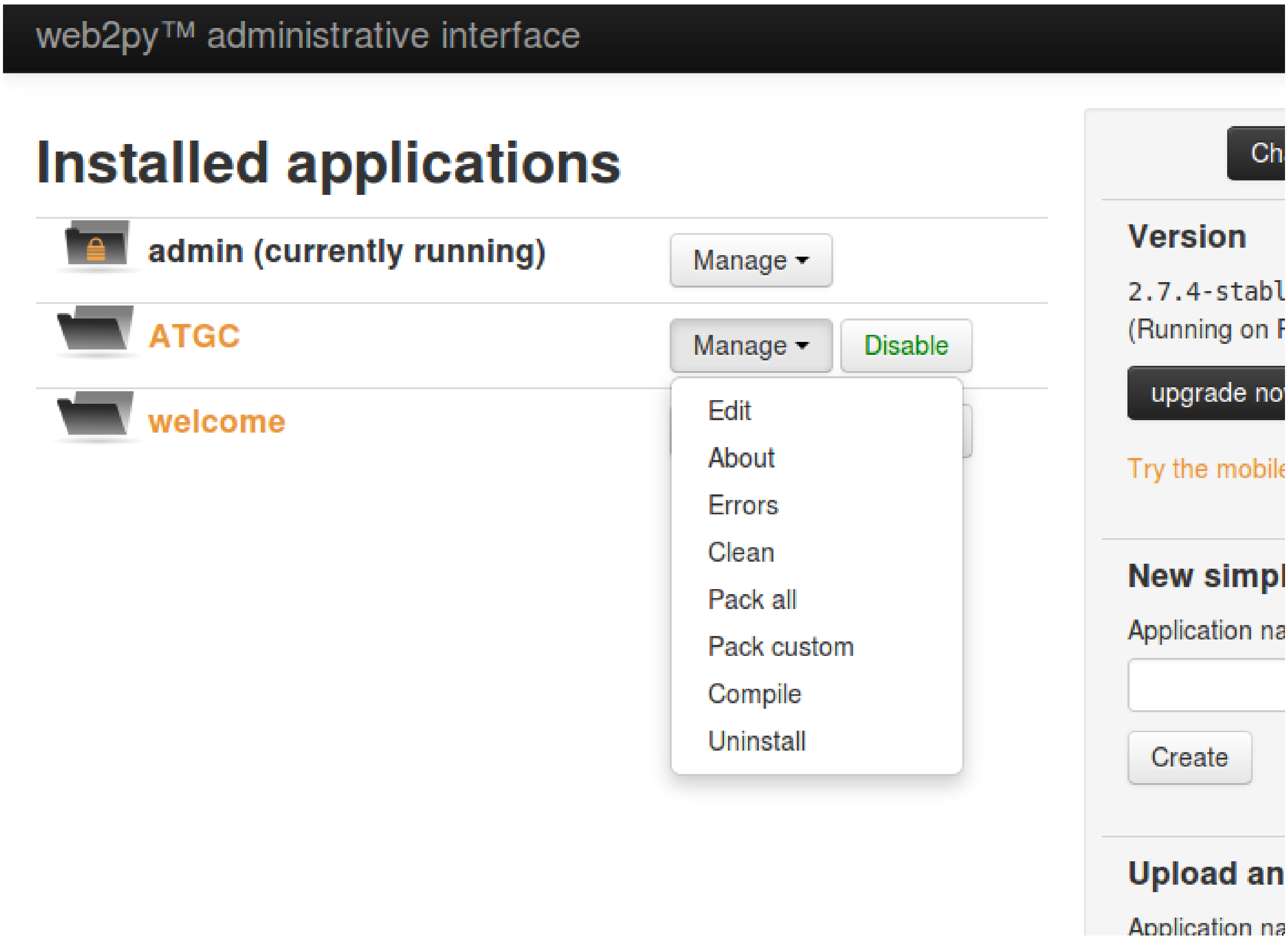

Figure 41: Choosing "Edit" option

Then, click in “database administration” button below of “Models” title

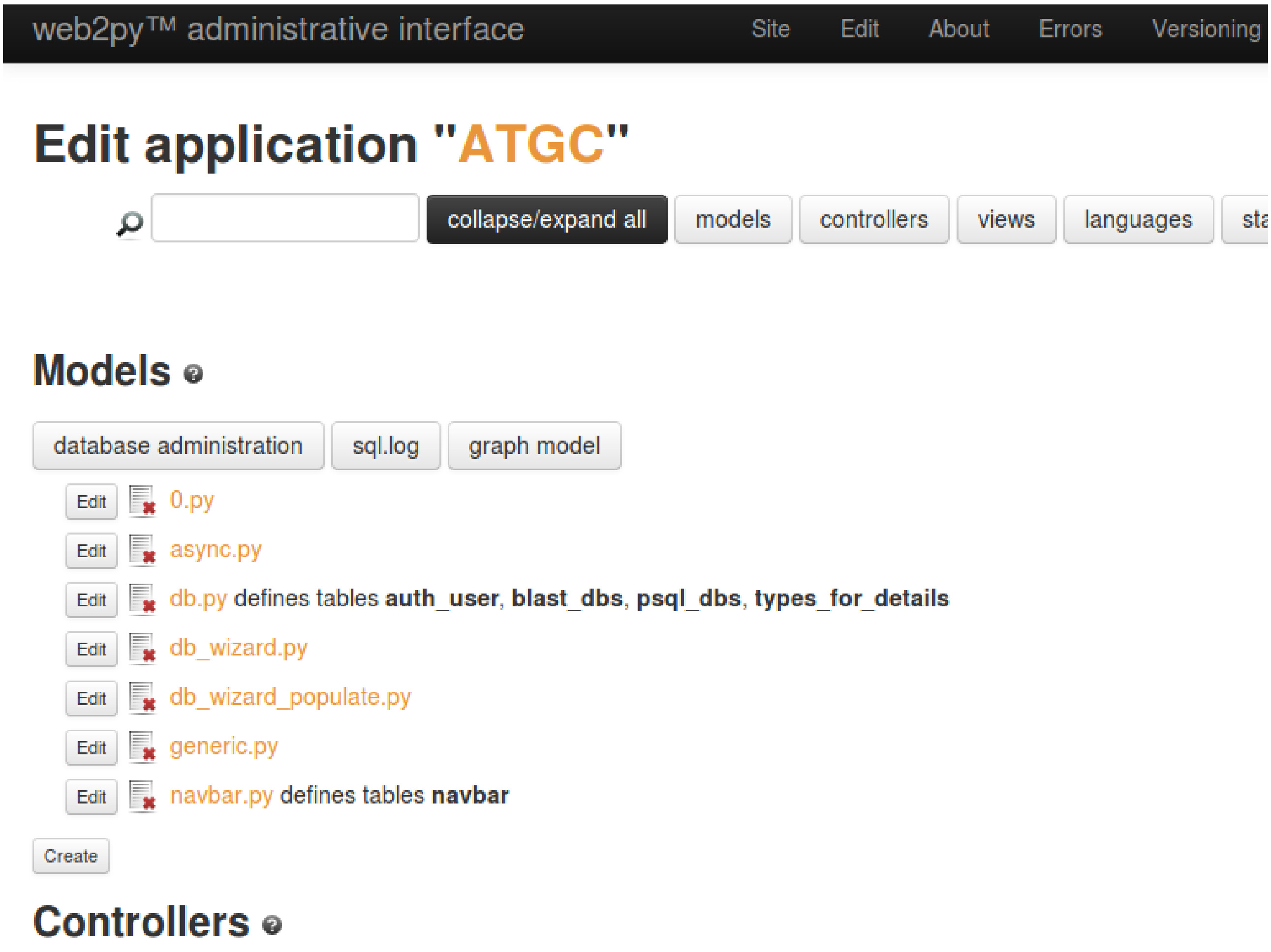

Figure 42: Click on "database administration" button

Choose the last option, db.navbar:

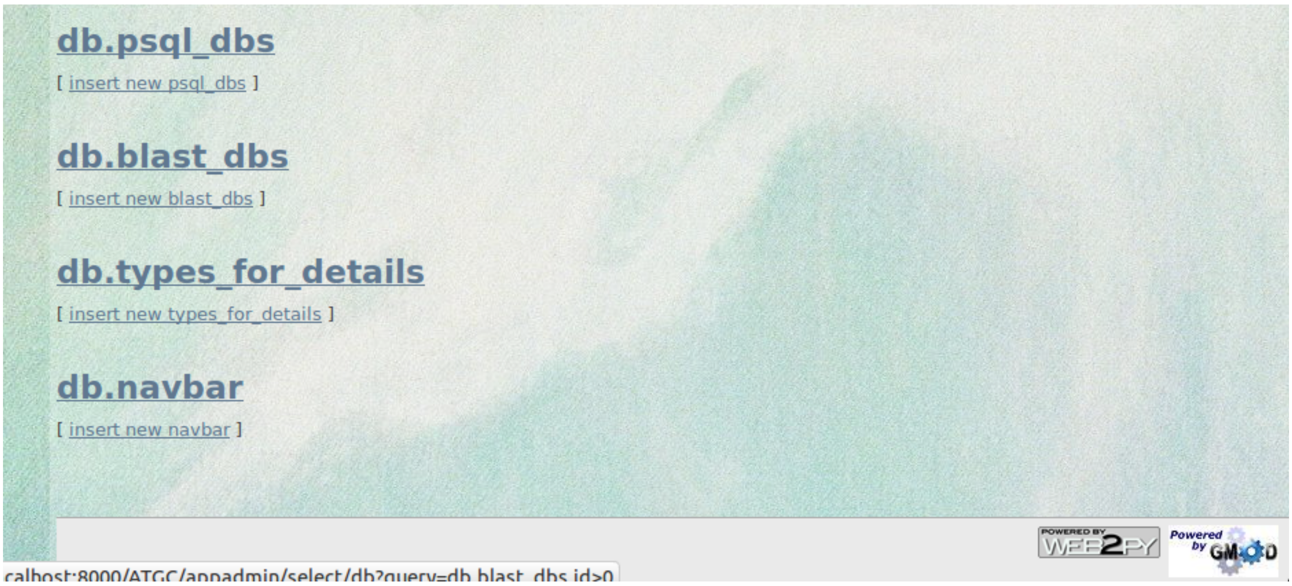

Figure 43: Click on db.navbar option

In the next page, you can see the table to set up the menu bar, at this point, you can choose which part of menu is visible or not, setting on “True” or “False” the field called “is_visible” for the correct table entry. For example, to hide the “Setup” menu you must complete the fields as the next figure:

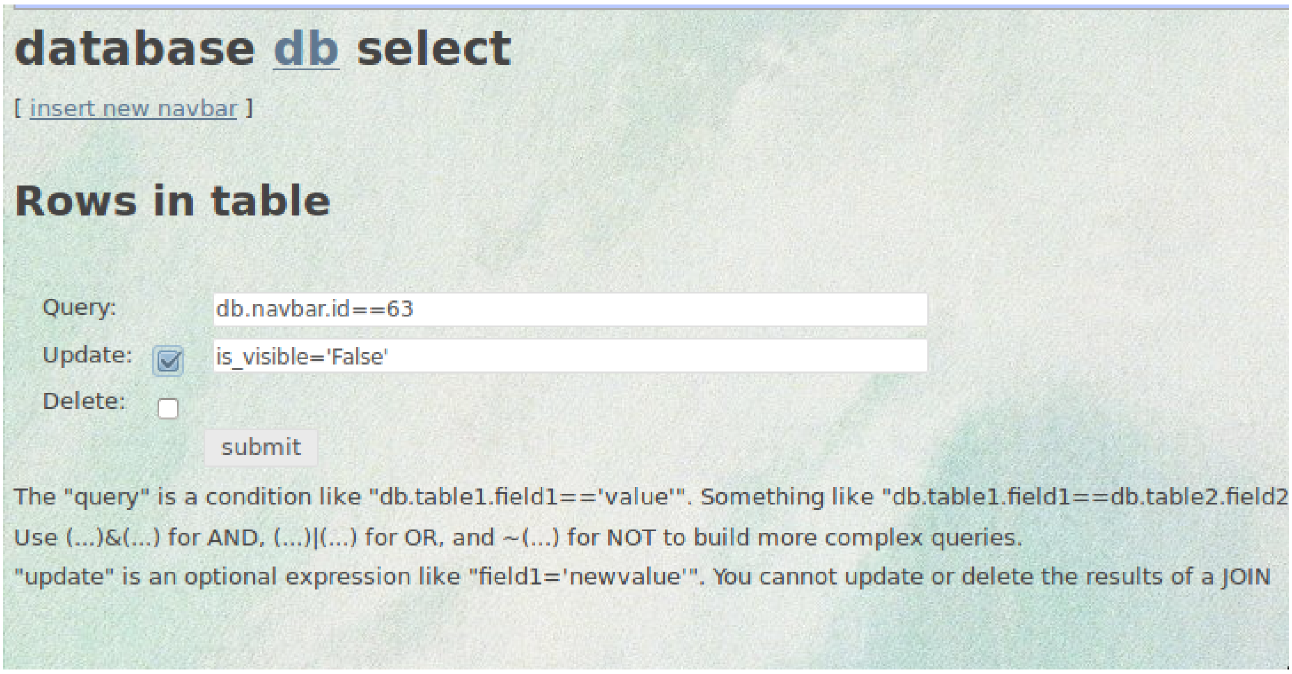

Figure 44: Hide the "Setup" part of the menu

For others main parts of the menu bar, the next table has a id value, you can hide any option of the menu, such as, complete part (for example, all Setup menu) or a single part (for example, only):

Menu option	Id (db.navbar)
Data Loading	2
Search	18
Ontology Annotation	23
Download	33
Software	36
Modify and Delete	42
Setup	63

Table 1: Id values for main menu bar options in db.navbar table

7 Testing the application

Inside the 'web2py/applications/ATGC/private/test' directory you have a test dataset to try the application, this dataset is composed by the following files:

- go.obo: Reduced gene ontology (obo format)
- so.obo: Complete sequence ontology (obo format)
- interpro2go: File with relationships between gene ontology and InterProScan terms
- test.png: Image to load when you create an organism
- transcripts.fasta: Multifasta file with sequences of 100 test transcripts
- transcripts.raw: Functional annotation results of InterProScan software
- transcripts.xml: Blast results
- transcripts.annot: Functional annotation results of Blast2GO software
- transcripts.gff3: Structural annotation of transcripts (gff3 format)
- genes.list: List of gene names to load with “Load features from list file (without sequence)”
- transcripts_gene.csv: csv file with relationships between genes and transcripts to load with “Feature relationships”
- transcripts_SNP.vcf: SNPs in transcripts
- transcripts_snps_line2.csv: Alleles of SNPs in line2
- transcripts_SSR.vcf: SSR in transcripts
- transcripts_ssrs_line2.csv: Alleles of SSRs in line2
- experiment_design.txt: Experiment design schema to load expression data (view 5.2.6 section in this tutorial) and create assay structure in the database
- lineX_control_Y.exp (X=1,2 and Y=1,2): RPKM expression values for control samples
- lineX_treated_X.exp (X=1,2 and Y=1,2): RPKM expression values for treated samples
