# Supplementary figures and images for: ATGC transcriptomics: a web-based application to integrate, explore and analyze de novo transcriptomic data
Source: BMC Bioinformatics. 2017 Feb 22;18:121. doi: 10.1186/s12859-017-1494-2 (PMC5320735; doi:10.1186/s12859-017-1494-2)

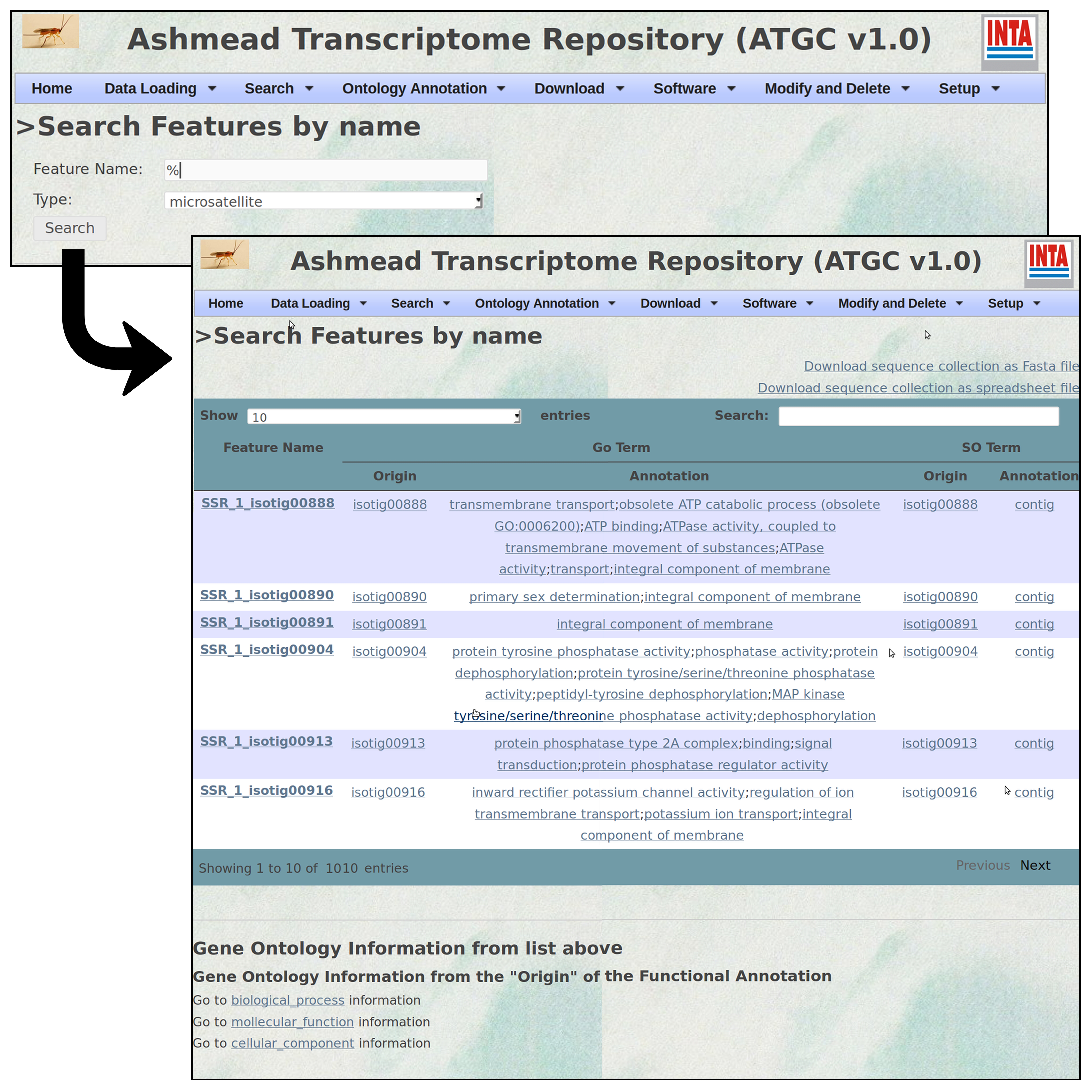

Supplement: Additional file 3: — Search strategy to obtain the complete list of microsatellites and results table containing isotigs associated to each molecular marker and your functional annotation. Below the table there are a three links to explore gene ontology information of the isotigs in the table. (JPG 9360 kb) [file 12859_2017_1494_MOESM3_ESM.jpg]

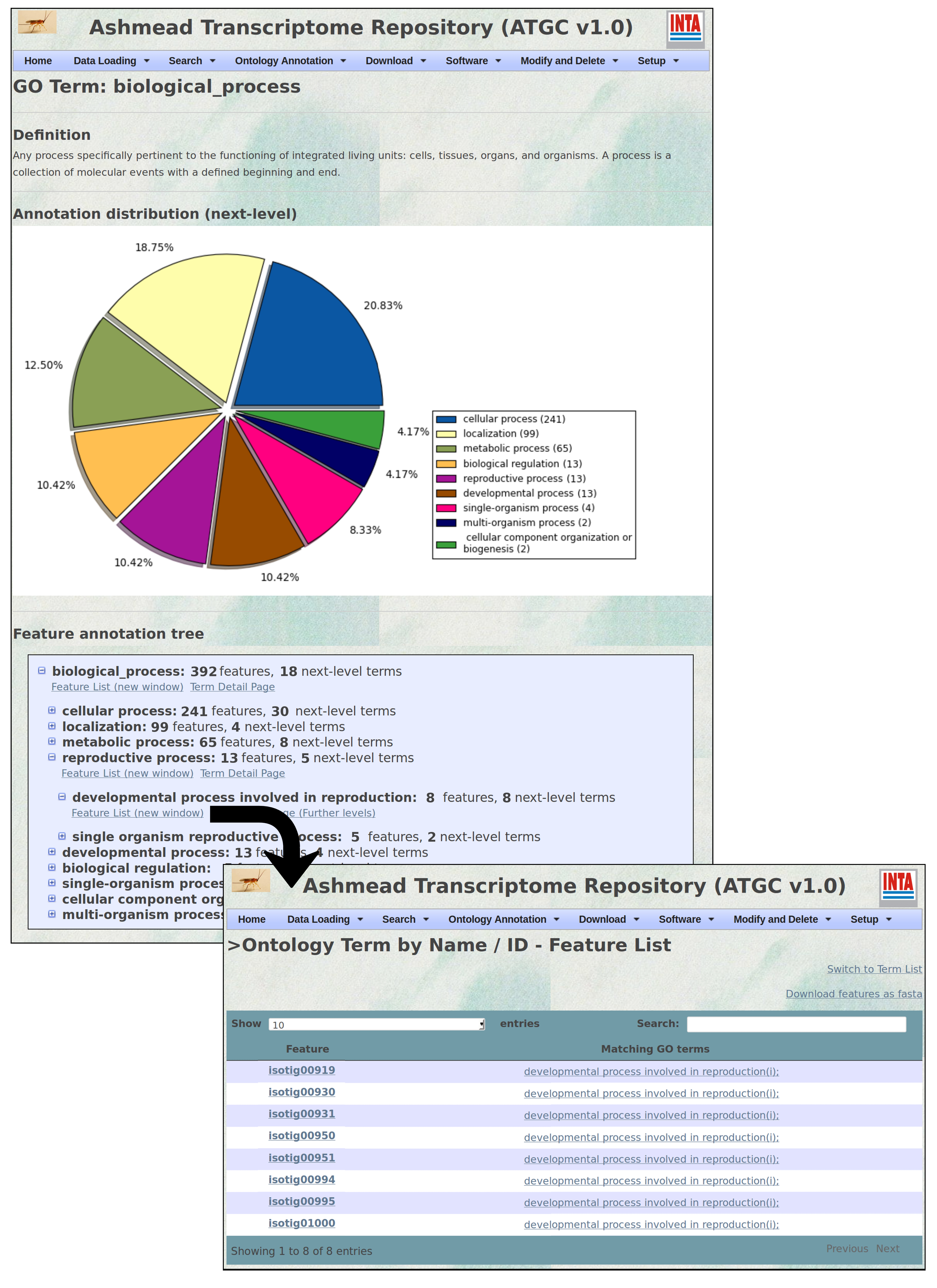

Supplement: Additional file 4: — Pie chart of “biological_process” GO term from links in Additional file 3. In the feature annotation tree, we explore until arrive to “developmental process involved in reproduction” term and go to obtain the list of features annotated with this term. (JPG 8198 kb) [file 12859_2017_1494_MOESM4_ESM.jpg]
